# Comparative Effects of Various Delivery Methods of Cognitive Behavioral Therapy for Insomnia Among Patients With Cancers: Systematic Review and Network Meta‐Analysis

**DOI:** 10.1002/pon.70593

**Published:** 2026-08-31

**Authors:** Chen‐I Lee, Muhammad Amirul Mukminin, Ya‐Wen Jan, Tzu‐Hao Wang, Tsai‐Wei Huang, Hsiao‐Yean Chiu

**Affiliations:** ^1^ School of Nursing College of Nursing Taipei Medical University Taipei Taiwan; ^2^ Department of Clinical Psychology Fu Jen Catholic University Taipei Taiwan; ^3^ Department of Psychology Chung Yuan Christian University Taoyuan Taiwan; ^4^ Cochrane Taiwan Taipei Medical University Taipei Taiwan; ^5^ Research Center in Nursing Clinical Practice Wan Fang Hospital Taipei Medical University Taipei Taiwan; ^6^ Research Center of Sleep Medicine College of Medicine Taipei Medical University Taipei Taiwan; ^7^ Research Center of Sleep Medicine Taipei Medical University Hospital Taipei Taiwan; ^8^ Department of Nursing Taipei Medical University Hospital Taipei Taiwan

**Keywords:** cancer, cognitive behavior therapy, insomnia, network meta‐analysis, sleep

## Abstract

**Background:**

Insomnia affects 30%–60% of cancer survivors, with around 20% meeting criteria for insomnia disorder. Cognitive behavioral therapy for insomnia (CBTi) is the first‐line treatment, but access is limited by cost and therapist availability, prompting exploration of alternative delivery modes, including digital interventions.

**Purpose:**

To systematically review and conduct a network meta‐analysis (NMA) of the efficacy of different CBTi delivery methods for managing insomnia in cancer survivors.

**Methods:**

The PubMed, Embase, CINAHL and Cochrane Library were searched for relevant articles published before September 25, 2025. Only randomized controlled trials of adult cancer patients evaluating the effects of any CBTi delivery mode on subjective sleep outcomes were included. Primary outcome was insomnia severity index (ISI) score; secondary outcomes included sleep onset latency (SOL), wake after sleep onset (WASO), total sleep time (TST), and sleep efficiency (SE). A frequentist random‐effects NMA was conducted.

**Results:**

Twenty‐two trials involving 2040 participants were included. Compared with treatment as usual (TAU), digital CBTi (dCBTi) demonstrated the largest reduction in ISI scores (MD = −9.70, 95% CI = −10.80 to −8.61), followed by telephone CBTi (MD = −8.74). dCBTi also significantly improved SOL (−30.95 min), WASO (−25.62 min), TST (+31.67 min), and SE (+14.63%). Group CBTi significantly improved SOL (−11.60 min) and WASO (−22.03 min). Meta‐regression analyses identified breast cancer diagnosis as a significant moderator of dCBTi effects on SOL (coefficient = 25.20, *p* = 0.015) and SE (coefficient = −16.17, *p* = 0.007). The certainty of evidence ranged from low to very low, primarily because of imprecision and network incoherence.

**Conclusions:**

dCBTi demonstrated the greatest potential for improving insomnia outcomes among the evaluated CBTi delivery modalities. However, given the low certainty of evidence and the presence of network inconsistency for selected outcomes, these findings should be interpreted cautiously. High‐quality head‐to‐head trials are needed to confirm the comparative effectiveness of different CBTi delivery modalities in oncology populations.

**Trial Registration:**

The study protocol was prospectively registered on PROSPERO (registration no. CRD42023429081)

## 1 Introduction

Cancer is one of the common causes of death around the world. The prevalence of insomnia is approximately 30%–60% in cancer survivors, with around 20% meeting the criteria for diagnosing insomnia, which is 2–3 times higher than the general population [1, 2, 3]. Insomnia can lead to fatigue [4, 5, 6], immune dysfunction [7], cognitive impairment [8, 9, 10, 11], prolonged hospitalization, reduced physical activity, and impaired quality of life [12, 13].

The American Academy of Sleep Medicine and the European Guideline for the Diagnosis and Treatment of Insomnia recommend cognitive behavioral therapy for insomnia (CBTi), a multicomponent approach, as the first‐line treatment for insomnia [14, 15, 16]. CBTi not only avoids adverse effects and dependency associated with pharmacological therapy but also shows sustained efficacy for both primary and comorbid insomnia [16, 17, 18]. However, the increasing demand for insomnia treatment, combined with therapist shortages, treatment costs, and geographical barriers, has limited the accessibility of conventional face‐to‐face CBTi [19, 20, 21]. To address these challenges, alternative delivery formats of CBTi have been increasingly explored to achieve comparable therapeutic efficacy while improving accessibility. In parallel, advances in digital technology and the growing acceptance of telehealth have accelerated the transition from traditional face‐to‐face interventions toward digitally delivered approaches [22].

In recent years, one network meta‐analysis (NMA) has examined the comparative efficacy of various CBTi delivery modes for cancer‐related insomnia [23]; however, the study categorized all control groups into two large groups: active and inactive control. The active group included various treatments (e.g., acupuncture, exercise, mindfulness‐based stress reduction), may violating the transitivity principle of NMA, which may lead to biased results and raise questions about the true comparative effectiveness between CBTi and other treatments. Furthermore, this NMA only included studies up to 2021, and recent publications should be considered. Therefore, an updated NMA with a more rational grouping approach is needed to compare the efficacy of different CBTi delivery methods in managing cancer‐related insomnia.

To the end, understanding which delivery mode had the optimal effects on insomnia alleviation may serve as a reference for future care guidelines, potentially advancing the accessibility of CBTi in clinical practice, particularly in cancer population. Therefore, to provide more appropriate recommendations and guidance for future technology‐driven clinical practice, this study aimed to conduct a NMA to evaluate the effectiveness of various CBTi delivery modes for cancer‐related insomnia.

## 2 Methods

This systematic review and network meta‐analysis was reported in accordance with the PRISMA extension for network meta‐analyses (PRISMA‐NMA) [24]. The details of checklist were listed in Supporting Information S1: Material 1. The review protocol was prospectively registered with PROSPERO (CRD42023429081).

### 2.1 Search Strategy

The PubMed, Embase, CINAHL and Cochrane Library were searched for relevant articles published before September 25, 2025. Keywords were conducted by using medical subject headings (MeSH) terms, text word searches and Boolean calculation. Search terms were developed around four key concepts: (1) cancers (e.g., “neoplasms”), (2) insomnia (e.g., “sleep initiation and maintenance disorders”, “sleep disorder”), (3) delivery modalities of CBT‐i (e.g., “telemedicine‐delivered”, online, “internet‐based”, “internet‐delivered”, “guided internet”, “web‐based”, “telephone‐based”, “telephone‐delivered”, mobile, “mobile‐application based”, “video‐based”, video, individual, “self‐administered”, group, “group‐delivered”), and (4) CBTi interventions (e.g., “cognitive behavio* therapy for insomnia” OR “CBT‐I” OR “behavio* therapy”). The details of search strategies were listed in Supporting Information S1: Material 2. Furthermore, we manually searched the reference lists of relevant studies for potential eligible studies. No publication languages or date was restricted.

### 2.2 Eligible Criteria

We included studies that (1) enrolled adult patients (≥ 18 years) with cancer diagnoses; (2) evaluated CBTi, delivered via any modality, as the experimental arm targeting insomnia; (3) adopted regimens aimed at alleviating insomnia, such as pharmacological treatment, aerobic exercise, Tai Chi Chuan, acupuncture, or usual care; (4) reported subjective sleep outcomes assessed by valid questionnaires (e.g., Insomnia Severity Index [ISI]) or sleep diaries; and (5) employed a randomized controlled trial (RCT) design. Studies were excluded if they were duplicate publications, conference abstracts, unavailable in full text, fail to provide extractable outcome data despite attempts to contact the original authors, or evaluated multicomponent interventions in which CBTi was combined with additional therapies.

### 2.3 Study Selection and Data Extraction

Two reviewers (C.I.L. and T.H.W) independently screened and cross‐checked the literature according to the inclusion and exclusion criteria to determine whether the article was eligible. Two reviewers used Endnote X20 to import all eligible studies. After removing the duplicate studies, two reviewers (C.I.L. and T.H.W) worked independently to screen the title and abstract of the studies. Then they reviewed the full text of each study based on the eligible criteria. Any disagreements were resolved by discussion or consultation a third reviewer (H.Y.C).

Two reviewers (C.I.L. and M.A.M.) independently extracted data, including outcome means and standard deviations. Discrepancies were resolved by consensus, with a third reviewer (H.Y.C.) adjudicating any remaining disagreements. The specific content of the characteristic data included basic information (i.e., first author, publication year), population information (i.e., number of patients, mean age, cancer stage), diagnosis tool, details of intervention (i.e., delivery method, length of treatment, content of the treatment), reference standards, and outcome indicators.

### 2.4 Assessment of Methodological Quality

The risk of bias of included RCTs was assessed using Cochrane risk‐of‐bias tool 2.0 [25]. Each study was evaluated across five domains: randomization process, deviations from intended interventions, missing outcome data, measurement of the outcome, and selection of the reported result. Each domain can be graded as “No,” “Probably No,” “Yes,” “Probably Yes,” “No Information,” or “Not Applicable.” An overall score can be classified into “Low”, “Some Concerns, or “High” risk based on the results of each domain. The independent researchers (C.I.L. and T.H.W) assessed the risk of bias in every study. Disagreements were resolved by discussion or consultation a third reviewer (H.Y.C).

### 2.5 Measurements of Treatment Outcomes

The primary outcomes contained insomnia severity measured using the ISI. It is a 7‐item self‐report questionnaire with scores ranging from 0 to 28, higher scores indicating more severe insomnia. The instrument has well‐established reliability and validity in adults (Cronbach's *α* = 0.85) [26]. Secondary outcomes consisted of subjective sleep parameters from sleep diary, including sleep onset latency (SOL), wake after sleep onset (WASO), total sleep time (TST), and sleep efficiency (SE).

### 2.6 Statistical Analysis

Results were presented as means and standard deviation (SDs) with 95% confidence intervals (CIs). If SDs could not be determined from the articles or original authors, we estimated them from *p* values and standard errors (SEs), or we imputed the SDs from an included trial which contain similar treatment groups [27]. If the SDs of post intervention values were not found, we imputed the change scores with corresponding SDs [28]. A frequentist framework with random‐effect NMA was conducted using the network commands for R. We created a network diagram at the treatment level to visualize the eligible studies. We conducted a forest plot to visualized individual effect and overall meta‐analysis effect. The overall effect difference was considered statistically significant if the *p* value was < 0.05.

For NMA, we generated league tables to demonstrate pairwise comparisons and p‐score to assess the relative ranking probabilities of interventions. P‐scores were interpreted as probabilities, with a higher p‐score (range, 0%–100%) indicating a greater likelihood of an intervention being ranked highly [29]. Statistical heterogeneity among studies was assessed using the Cochran's *Q* test, with *p* < 0.10 indicating potential heterogeneity. The I^2^ statistic, with values of 25%, 50%, and 75% representing low, moderate, and high heterogeneity, indicates the degree of between‐study heterogeneity [30]. Inconsistencies were estimated by using local (node‐splitting) and global (design‐by‐treatment interaction) models [31]. To address potential heterogeneity or inconsistency, we performed meta‐regression analyses to explore moderating effects on treatment outcomes. The moderators included cancer type (breast cancer vs. non‐breast cancer), treatment phase (active treatment vs. post‐treatment), and inclusion criteria of insomnia (broad definition vs. rigor definition). Additionally, sensitivity analysis was further performed by excluding the study adopted imputed standard deviation to examine the robustness of our findings. Egger's test was performed to assess potential publication bias, with statistical significance set at *p* < 0.05 [32].

The certainty of evidence for each pairwise comparison within the network meta‐analysis was systematically evaluated using the CINeMA (Confidence in Network Meta‐Analysis) framework [33]. The assessment was performed via the official CINeMA web interface, which operationalizes the evaluation across six methodological domains: within‐study bias, reporting bias, indirectness, imprecision, heterogeneity, and incoherence [34]. Based on the semi‐automated integration of study‐level characteristics and network structure provided by the platform, the overall confidence for each primary outcome was consensus‐rated and categorized into four levels: high, moderate, low, or very low [33].

## 3 Results

### 3.1 Selection, Inclusion, and Characteristics of Studies

**FIGURE 1:**
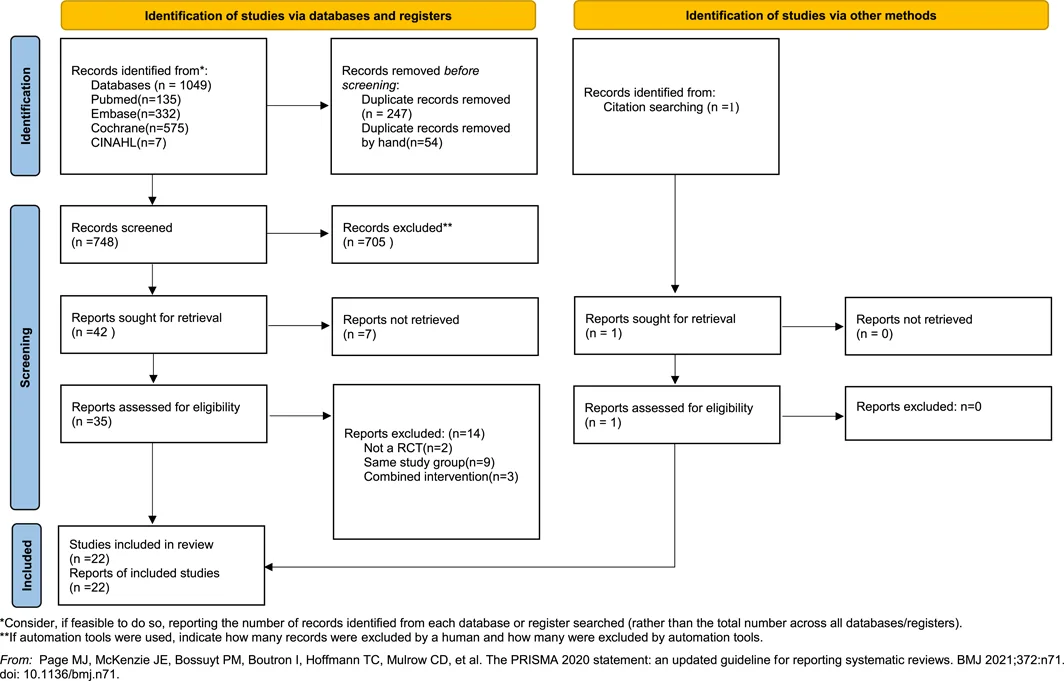
PRISMA flow diagram.

A total of 1049 potential articles were identified in the initial search. After excluding the duplicates and irrelevant articles, the remaining 35 articles were selected for full‐text screening. Of these, 14 articles were further excluded for the following reasons: (1) studies were not RCTs (*n* = 2); (2) studies involved overlapping participant populations (*n* = 9); and (3) interventions consisted of multi‐component or combined modalities that did not meet predefined inclusion criteria (*n* = 3; Supporting Information S1: Material 3). One study was additionally identified from relevant citation searching, leading to 22 articles fulfilling the inclusion criteria (Figure 1).

The general information of included articles is presented in Table 1, while a detailed summary of study characteristics is provided in Supporting Information S1: Material 4. A total of 22 eligible RCTs, comprising 2040 participants with a mean age of 56.1 years, were included. Of these, 10 trials exclusively enrolled patients with breast cancer, whereas remaining 12 involved mixed cancer populations, predominantly including prostate, hematological, and gynecological cancers. Clinically, most study (20/22, 90.9%) were conducted during the post‐treatment stage. Four approaches were used to define insomnia eligibility: (1) symptom‐based screening using a single self‐report scale (ISI ≥ 8 or Pittsburgh sleep quality index > 5); (2) hybrid criteria combining formal clinical diagnoses (DSM‐V/ICSD‐III) with questionnaire cut‐off scores; (3) quantitative parameters from prospective sleep diaries (SOL or WASO > 30 minutes, frequency ≥ 3 nights/week, with or without sleep efficiency < 85%); and (4) psychiatric diagnoses established exclusively via structured clinical interviews (DSM‐IV/V, ICSD‐II/III). Regarding the interventions, the total duration of CBTi predominantly ranged from 6 to 8 weeks (6 weeks being the most prevalent, 13/22, 59%). Session lengths varied by format, lasting 50–90 minutes for face‐to‐face or synchronous video sessions, and 35–60 minutes for digital modules.

**TABLE 1:** The characteristics of included studies.

Author (year)	Cancer type	Cancer stage	Diagnosis tool of insomnia	CBTi, n	Control group(*n*)	Mean age	Component of CBTi	Duration/frequency and other detail of CBTi	Follow‐up	Subjective outcome measurement
Casault (2015) [35]	Mixed cancer	Not mention	ISI≥ 8	sCBTi,17	TAU, (18)	56.9	SC,SR,SH/SE,CR	6 wks, 6 short booklets+3 phone consultations with a psychologist (20 min)	Pre, post, 3m, 6m	ISI [sleep diary] SOL, WASO, SE(%), TST, TWT
Clara (2025) [36]	Mixed cancer, who completed active treatment	Mixed	ISI≥ 8	**dCBTi**, 70	TAU, 68	47	SC, SR, SH/SE, CR	6 weekly online modules + email clinician	Pre, post	ISI [sleep diary] TST, SE
Dirksen (2008) [37]	BC survivors, completed cancer treatment≥ 3m	Stage I‐III	DSM‐IV‐TR, ICSD	iCBTi, 40	SH, 41	58.3	SC,SR, SH/SE,	6 wks, in‐person classes (1 or 2 h) or 15 min by‐phone session	Pre, post	ISI
Espie (2008) [38]	Mixed cancer	Not mention	DSM‐IV‐TR, ICSD	gCBTi, 100	TAU, 50	61	SC,SR,CR	5 session weekly	Pre, post, 6m	[Sleep diary] SOL, WASO, TST, SE(%)
Fiorentino (2010) [39]	BC survivors, completed BC treatment	Stage I‐III	DSM‐IV	iCBTi, 11	TAU, 10	61	SR, SC, SH/SE, RT, CR	6 weekly individual CBTi sessions	Pre, post, 6 wks	ISI [sleep diary] TST, WASO, awakenings, SE (%)
Garland (2014) [40]	Mixed cancer	Nonmetastatic cancer	DSM‐IV, ICSD‐2	gCBTi, 47	MBSR, 64	58.9	SC, SR, CR, RT	8 weekly, 90‐ min sessions, for a total of 12 contact hours, in groups of 6–10 patients	Pre, post, 5m	ISI, [sleep diary]
Garland (2019) [41]	Mixed cancer, completed active treatment at least 1m	Not mention	ISI > 7, DSM‐V	iCBTi, 80	Acupuncture, 80	61.5	SC,SR,CR, RT, SH	8 individual sessions, 5 weekly followed by biweekly session(first session 60 mins, remaining 30 mins)	Pre, post, 3m	ISI [sleep diary] SOL, WASO, TST, SE(%)
Garland (2024) [42]	Mixed cancer	NC	ISI≥ 8	tCBTi, 63	TAU, 69	NC	SC, SR, SH/SE, CR	7 weekly live video 1:1 with trained PHD researchers	Post‐treatment, 3‐ & 6‐month follow‐up	ISI
Hall (2021) [43]	Mixed cancer, completed active treatment	Nonmetastatic cancer	DSM‐V, ISI≥ 15	tCBTi, 20	SH, 20	53.2	SC,SR,CR, RT, SH	4 weekly individual synchronous 45 mins sessions	Pre, post, 1m	ISI, PSQI [sleep diary] SOL, WASO, TST, SE(%)
Irwin (2017) [44]	BC survivor, completed BC treatment	Not mention	DSM‐IV‐TR, DSM‐V, ICSD‐2	gCBTi, 45	EX, 45	60	SC, SH, SR, RT, CR	CBTi teaching 2 months + 1 month of skill consolidation and adherence; groups of 7–10 participants in weekly 120 min sessions	Pre, mid, post, 3 m, 12 m	[Sleep diary] SOL, WASO, TST, SE (%)
Matthews (2014) [45]	BC survivors, completed BC treatment	Stage I‐III	ISI≥ 8, IIS	iCBTi, 32	BT, 28	52	SR, SC, SH/SE, CR	6 weekly individual, in person CBTi sessions (30–60 min each) or by phone (15–20 min each)./BPT: SH/SE	Pre, post, 3 m, 6 m	ISI [sleep diary] SOL, WASO, TST, awakenings, SE (%)
Mercier (2018) [46]	Mixed cancer, completed active treatment at least 6m	Nonmetastatic cancer	ISI≥ 8	sCBTi, 21	EX, 20	57.1	SC, SR, SH/SE, CR	6 week individual sessions, 60‐min DVD+ 6 booklets, weekly phone call from researcher	Pre, post, 3 m, 6 m	ISI [sleep diary] SOL, WASO, TST, awakenings, SE (%)
Moon (2020) [47]	Mixed cacncer, completed direct treatment for a month to 5 years	Stage I‐III	ISI≥ 8	Herb, 11	iCBTi, 11	63	SC, SR, SH, CR, RT	CBTi: 4 weekly session + SH handout/Cheonwangbosimdan: Once a day for 4 weeks	Pre, post	ISI
Oswald (2022) [48]	BC patient completed radio‐/chemotherapy or surgery	no mention	ISI≥ 8	tCBTi, 15	TAU, 15	58.4	SC, SR, SH/SE, CR, relapse prevention, hypnotic usage	Virtual group CBTi: 6weekly (90 min) videoconference, in group of 10 participant	Pre, post	ISI [sleep diary] SE(%)
Ritterband (2011) [49]	Mixed cancer, completed active treatment	Stage I‐IV	DSM‐IV‐TR + symptom criteria	**dCBTi**, 14	TAU, 14	56.7	SC, SR, SH/SE, CR, relapse prevention	6–9 wks, including 6 wks online cores(45–60 min)	Pre, post	ISI [sleep dairy] SOL,WASO, TIB, TST, awakenings, SE(%)
Savard (2005) [50]	BC survivors, completed radio‐/chemotherapy	Stage I‐III	DSM‐IV, ICSD,	gCBTi, 27	TAU, 30	54.1	SC, SR, CR, SH/SE, fatigue and stress management	8 weekly 90 min sessions, one booster session, in groups of 4–6 patients	Pre, post, 3 m, 6 m, 12 m	ISI [sleep diary] SE (%), TWT, TST, SOL, WASO
Savard (2014) [51]	BC patients, with radiation therapy within the past 18 m	Stage 0‐III	ISI≥ 8 or psychotropic medication as a sleep aid 2 nights in the past 2 wks	iCBTi, 81/sCBTi, 80	TAU, 81	54.6	SC, SR, SH	6 wks of individual CBTi: Weekly, 50 min session video CBTi: Video segment (5–20 min each) with booklets each week	Pre, post, 3 m, 6 m, 12 m	ISI [sleep diary] SOL, WASO, EMA, TWT, TST, SE (%)
Savard (2021) [52]	Mixed cancer	Nonmetastatic cancer	ISI≥ 8	iCBTi, 59	Stepped‐care CBTi, 1)web‐based, 65 2) face to face, 53	55.2	SC, SR, SH/SE, CR	CBTi: 6 weekly face ‐to‐face sessions(50 min) + booklet. Step CBTi: First step: web‐based for less severe ISI, face‐t0‐face for more severe ISI. Second step: ISI< 8: no further treatment, ISI > =8: Three 50‐min booster sessions of face‐to‐face individual CBTi every 2 week	Pre, post, 6m, 12m	ISI [sleep diary] SOL, WASO, TWT, TST, SE(%)
Starling (2024) [53]	BC survivors	Mixed	ISI > 8	**dCBTi**, 38	sCBTi, 38	61.2	SC, SR, SH/SE, CR	Self‐guided CBTi: Fully automated Voice‐delivered, self‐guided CBTi, personalize suggestion based on sleep diary.	Pre, post	ISI [sleep diary] SOL, WASO, TWT, TST, SE(%)
Tian (2025) [54]	Mixed	Nonmetastatic cancer	DSM‐V, ICSD‐III, ISI≥ 14	iCBTi, 23	rTMS,22/sham‐rTMS, 21		SC, SR, SH/SE, CR	Individual CBTi: 6 weekly 90 min sessions face‐to‐face CBTi. rTMS: Non‐invasive neuromodulation technique that delivers repeated magnetic pulses to targeted areas of the brain to influence neural activity. Sham‐rTMS: To mimic the sensory experience of real rTMS without delivering actual magnetic stimulation.	Pre, post	ISI
Zachariae (2018) [55]	BC patients	Not mention	PSQI > 5	sCBTi, 133	TAU, 122(after 15 wks)	53.1	SC, SR, SH/SE, CR	Self administered online 6‐cores session (6–9 wks, 45–50 min each)	Pre, post, 15 wks	ISI, PSQI [sleep diary] SOL, WASO, TST, SE(%)
Zheng (2015) [56]	BC patient	Stage I‐III	ICSD, DSM‐IV	gCBTi. 45	TAU, 48	NC	SC, SR, SH/SE, CR	Group CBTi, 8 weekly session, first session 2 hrs, the following session 1 hr each time, in group of 6–10 people + booklet	Pre, post	ISI [sleep diary] SOL, WASO, TST, SE

Abbreviations: BC = breast cancer, BT = behavioral therapy, CBTi = cognitive behavioral therapy for insomnia, CC = component control, CR = cognitive reconstructing, dCBTi = digital cognitive behavioral therapy, DSM = Diagnostic and Statistical Manual of Mental Disorders, DSM‐IV = Diagnostic and Statistical Manual of Mental Disorders, Fourth Edition, DSM‐IV‐TR = Diagnostic and Statistical Manual of Mental Disorders, Fourth Edition, Text Revision, EX = aerobic exercise, gCBTi = group cognitive behavioral therapy, iCBTi = individual cognitive behavioral therapy,  ICSD = International Classification of Sleep Disorders by the Diagnostic and Statistical Manual, ISI = insomnia severity index, MBSR = Mindfulness‐Based Stress Reduction, NC = not clear, NT = no treatment, PSQI = Pittsburgh Sleep Quality Index, RT = relaxation training, rTMS = repetitive transcranial magnetic stimulation, SC = sleep control, sCBTi = self help cognitive behavioral therapy, SE = sleep education, sleep efficiency, SH = sleep hygiene, SOL = sleep onset latency, SR = sleep restriction, TAU = treatment as usual, tCBTi = tele cognitive behavioral therapy, TST = total sleep time, TWT = total wake time, WASO = wake after sleep onset.

Definition of the included regimens are presented in Table 2. A total of 14 treatment modalities were identified in the NMA. CBTi interventions were categorized into six groups based on their delivery modes: digital cognitive behavioral therapy (dCBTi), group cognitive behavioral therapy (gCBTi), individual cognitive behavioral therapy (iCBTi), self‐help cognitive behavioral therapy (sCBTi), stepped‐care cognitive behavioral therapy (Stepcare), and tele cognitive behavioral therapy (tCBTi). Noted that Stepcare was defined as a sequential delivery model in which intervention intensity was escalated from self‐help approaches to individualized therapy based on patient needs. Although Stepcare incorporated multiple components, it was treated as a single delivery trajectory within an individual study to evaluate its overall efficacy [52]. Non‐CBTi comparators were grouped based on their primary therapeutic components and underlying physiological mechanisms, including acupuncture, behavioral therapy (BT), exercise (EX), herb, mindfulness‐based stress reduction (MBSR), repetitive transcranial magnetic stimulation (rTMS), sleep hygiene (SH), and treatment as usual (TAU).

**TABLE 2:** The definition of study interventions.

Grouping name	Definition
Acupuncture (acupuncture)	A component of traditional Chinese medicine in which an acupuncturist inserts needles at specific locations on the body. Included treatment: Acupuncture.
Behavioral placebo (BT)	A brief behavioral intervention targeting arousal‐reducing behaviors such as avoiding stimulants or maintaining a regular bedtime. Included treatment: Behavioral placebo treatment.
Digital cognitive behavioral therapy (dCBTi)	Internet‐ or app‐based CBTi programs deliver the same core components via digital modules. Allow self‐paced learning with automated feedback. Included treatment: App, automated interaction CBTi.
Exercise (EX)	Aerobic exercise increases the body's demand for oxygen, thereby adding to the workload of the heart and lungs, and raising the heart rate. Included treatment: Tai chi, aerobic exercise.
Group cognitive behavioral therapy (gCBTi)	Face‐to‐face CBTi sessions in groups focusing on restructuring the thoughts, feelings, and behaviors that are contributing to insomnia. Core components include sleep restriction, stimulus control, cognitive therapy, sleep hygiene education, and relaxation training. Included treatment: group CBTi.
Herb	Use of herbal remedies is purported to improve sleep through sedative or anxiolytic effects. Included treatment: Cheonwangbosimdan.
Individual cognitive behavioral therapy (iCBTi)	Face‐to‐face individual CBTi focuses on restructuring the thoughts, feelings, and behaviors that are contributing to insomnia. Core components include sleep restriction, stimulus control, cognitive therapy, sleep hygiene education, and relaxation training. Included treatment: Individual CBTi.
Mindfulness‐based stress reduction (MBSR)	Meditation techniques and gentle yoga are practiced supporting the development of mindful awareness and responding to stress. Included treatment: MBSR.
Repetitive transcranial magnetic stimulation (rTMS)	Non‐invasive neuromodulation targets specific brain regions to influence sleep regulation. Included treatment: rTMS.
Self‐help cognitive behavioral therapy (sCBTi)	CBTi without therapist interaction. Usually provided through booklets, websites, or videos to educate patients. Core components include sleep restriction, stimulus control, cognitive therapy, sleep hygiene education, and relaxation training. Included treatment: Online, video, booklet CBTi.
Sleep hygiene (SH)	Education on lifestyle and environmental factors affecting sleep, including regular sleep schedules, caffeine reduction, and bedroom optimization.
Stepped‐care cognitive behavioral therapy (stepcare)	Based on patient insomnia severity to provide an optimal CBT‐I delivery method (including iCBTi and sCBTi) Core components include sleep restriction, stimulus control, cognitive therapy, sleep hygiene education, and relaxation training. Included treatment: Stepped‐care CBTi.
Tele cognitive behavioral therapy (tCBTi)	CBTi conducted via video or phone sessions, maintaining core components remotely. Ensures accessibility while preserving therapist guidance. Included treatment: Virtual, synchronous CBTi
Treatment as usual (TAU)	Standard clinical care or routine treatment provided in the absence of a structured insomnia intervention. Included treatment: TAU, waiting list, no treatment.

### 3.2 Risk of Bias

Of 22 included studies, seven studies were judged as overall low risk, whereas 10 were judged as having some concerns and five as being at high risk of bias. The primary methodological concerns arose from the lack of blinding of study personnel, post‐randomization exclusions, and differential attrition across treatment arms. The summary of risk of bias is shown in Supporting Information S1: Material 5.

### 3.3 Network Plots

**FIGURE 2:**
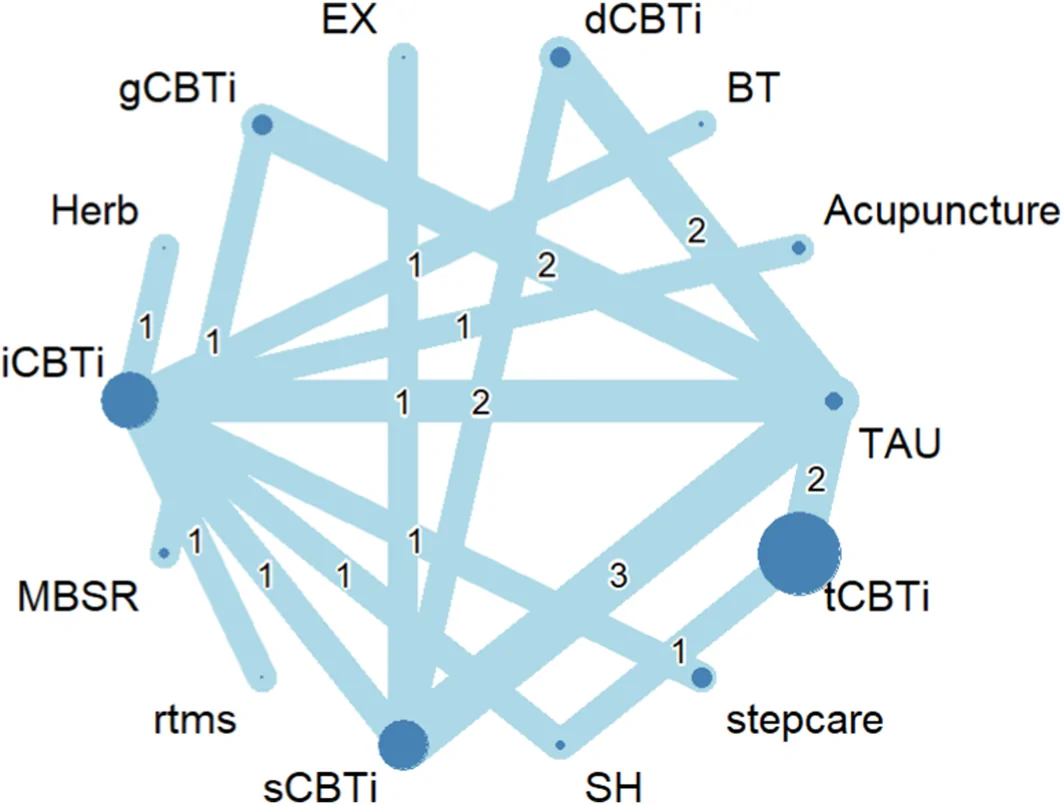
Network map for insomnia severity. BT = behavioral therapy, dCBTi = digital CBTi, EX = exercise, gCBTi = group CBTi, iCBTi = individual CBTi, MBSR = mindfulness‐based stress reduction, rTMS = repetitive transcranial magnetic stimulation, sCBTi = self‐help CBTi, SH = sleep hygiene, stepcare = stepped‐care CBTi, TAU = treatment as usual, tCBTi = teleCBTi and TST = total sleep time.

Network plot of primary and secondary outcomes are illustrated in Figure 2 and Supporting Information S1: Material 6. For the ISI, 14 interventions consisting of 19 trials were presented. Meanwhile, 10 interventions from 15 trials were included for SOL, WASO, and TST, while SE featured 12 interventions across 17 trials. The number of participants in each plot was between 1301 and 1511. Two closed loops were formed in each sleep outcome. Among the node comparisons, TAU was the most compared arm.

### 3.4 Effects of Various Delivery Modes of CBTi on Insomnia

For primary outcome (ISI score), 19 studies were included, of which eight focused on breast cancer, while the remaining studies involved mixed cancer populations (e.g., prostate cancer, colorectal cancer, lung cancer and ovarian cancer). As shown in Table 3, relative to TAU, dCBTi showed the greatest improvement in ISI scores (mean difference = −9.70, 95% CI: −10.80 to −8.60), followed by tCBTi (−8.74, −10.05 to −7.42), herb (−7.61, −12.08 to −3.13), sCBTi (−5.34, −6.33 to −4.35), gCBTi (−5.06, −5.64 to −4.48) and iCBTi (−4.61, −6.48 to −2.74). Furthermore, dCBTi demonstrated significantly greater reductions in ISI scores compared with all CBTi delivery modes and non‐CBTi regimens (i.e., acupuncture, BT, EX, MBSR, rTMS, SH, TAU), with exception of the comparisons with tCBTi and herb. P‐score ranking further indicated that dCBT‐I and tCBT‐I had the highest probabilities of being ranked the two most effective interventions (0.975 and 0.908, respectively; Supporting Information S1: Material 7).

**TABLE 3:** League table of treatment effects on insomnia severity index scores.

dCBTi	.	.	−5.30 (−7.57 to −3.03)	.	.	.	.	.	.	.	.	.	−9.43 (−10.65 to −8.21)
−0.97 (−2.68 to 0.75)	tCBTi	.	.	.	.	.	.	.	−5.41 (−8.18 to −2.65)	.	.	.	−8.83 (−10.21 to −7.44)
−2.09 (−6.70 to 2.51)	−1.13 (−5.70 to 3.44)	Herb	.	.	−3.00 (−7.06 to 1.06)	.	.	.	.	.	.	.	.
**−4.36 (−5.68 to −3.04)**	**−3.39 (−5.04 to −1.75)**	−2.27 (−6.84 to 2.31)	sCBTi	.	2.00 (−3.42–7.42)	−1.80 (−5.99 to 2.39)	.	.	.	.	.	.	−5.56 (−6.63 to −4.49)
**−4.64 (−5.88 to −3.40)**	**−3.68 (−5.12 to −2.24)**	−2.55 (−7.06 to 1.96)	−0.28 (−1.43 to 0.86)	gCBTi	.	.	.	.	.	.	.	−3.78 (−5.38 to −2.18)	−5.06 (−5.64 to −4.48)
**−5.09 (−7.26 to −2.93)**	**−4.13 (−6.22 to −2.04)**	−3.00 (−7.06 to 1.06)	−0.73 (−2.83 to 1.37)	−0.45 (−2.41 to 1.51)	iCBTi	.	−1.20 (−2.60 to 0.20)	−1.30 (−5.42 to 2.82)	−1.93 (−4.33 to 0.47)	−2.34 (−4.96 to 0.28)	−2.60 (−4.09 to −1.11)	.	−4.28 (−6.42 to −2.15)
**−6.16 (−10.56 to −1.76)**	**−5.19 (−9.70 to −0.69)**	−4.07 (−10.27 to 2.14)	−1.80 (−5.99 to 2.39)	−1.52 (−5.86 to 2.83)	−1.07 (−5.76 to 3.62)	EX	.	.	.	.	.	.	.
**−6.29 (−8.87 to −3.71)**	**−5.33 (−7.84 to −2.81)**	−4.20 (−8.50 to 0.10)	−1.93 (−4.46 to 0.59)	−1.65 (−4.06 to 0.76)	−1.20 (−2.60 to 0.20)	−0.13 (−5.03 to 4.76)	Stepcare	.	.	.	.	.	.
**−6.39 (−11.05 to −1.74)**	**−5.43 (−10.05 to −0.81)**	−4.30 (−10.09 to 1.49)	−2.03 (−6.66 to 2.59)	−1.75 (−6.31 to 2.82)	−1.30 (−5.42 to 2.82)	−0.23 (−6.48 to 6.01)	−0.10 (−4.45 to 4.25)	rtms	.	.	.	.	.
**−6.75 (−9.23 to −4.27)**	**−5.78 (−7.95 to −3.61)**	**−4.65 (−9.19 to −0.12)**	−2.39 (−4.81 to 0.04)	−2.10 (−4.40 to 0.20)	−1.65 (−3.67 to 0.37)	−0.59 (−5.43 to 4.26)	−0.45 (−2.91 to 2.00)	−0.35 (−4.95 to 4.24)	SH	.	.	.	.
**−7.43 (−10.83 to −4.03)**	**−6.47 (−9.82 to −3.12)**	**−5.34 (−10.17 to −0.51)**	−3.07 (−6.43 to 0.28)	−2.79 (−6.06 to 0.48)	−2.34 (−4.96 to 0.28)	−1.27 (−6.65 to 4.10)	−1.14 (−4.11 to 1.83)	−1.04 (−5.92 to 3.84)	−0.69 (−3.99 to 2.62)	BT	.	.	.
**−7.69 (−10.32 to −5.06)**	**−6.73 (−9.29 to −4.16)**	**−5.60 (−9.93 to −1.27)**	**−3.33 (−5.91 to −0.76)**	**−3.05 (−5.51 to −0.59)**	**−2.60 (−4.09 to −1.11)**	−1.53 (−6.46 to 3.39)	−1.40 (−3.45 to 0.65)	−1.30 (−5.68 to 3.08)	−0.95 (−3.46 to 1.57)	−0.26 (−3.27 to 2.75)	Acupuncture	.	.
**−8.42 (−10.45 to −6.40)**	**−7.46 (−9.61 to −5.31)**	**−6.33 (−11.12 to −1.55)**	**−4.06 (−6.03 to −2.10)**	**−3.78 (−5.38 to −2.18)**	**−3.33 (−5.86 to −0.80)**	−2.26 (−6.90 to 0.37)	−2.13 (−5.02 to 0.76)	−2.03 (−6.87 to 2.81)	−1.68 (−4.48 to 1.12)	−0.99 (−4.63 to 2.65)	−0.73 (−3.67 to 2.20)	MBSR	.
**−9.70 (−10.80 to −8.60)**	**−8.74 (−10.05 to −7.42)**	**−7.61 (−12.08 to −3.13)**	**−5.34 (−6.33 to −4.35)**	**−5.06 (−5.64 to −4.48)**	**−4.61 (−6.48 to −2.74)**	−3.54 (−7.85 to 0.77)	−3.41 (−5.75 to −1.07)	−3.31 (−7.84 to 1.22)	−2.95 (−5.18 to −0.73)	−2.27 (−5.49 to 0.95)	−2.01 (−4.40 to 0.39)	−1.28 (−2.98 to 0.42)	TAU

*Note:* Values in bold indicate significant. The cells in the upper‐right section display the results of direct comparisons among interventions, while the cells in the lower‐left section represent the network estimates combining both direct and indirect evidence. All outcomes are reported as Mean Differences (MD) with 95% confidence intervals (CI), where values in bold indicate statistically significant differences (*p* < 0.05). For the ISI score changes, a negative value (MD < 0) demonstrates that the intervention in the top row achieved a greater reduction in insomnia severity than the intervention in the right‐hand column.

Abbreviations: BT = behavioral therapy, dCBTi = digital CBTi, EX = exercise, gCBTi = group CBTi, iCBTi = individual CBTi, MBSR = mindfulness‐based stress reduction, rTMS = repetitive transcranial magnetic stimulation, sCBTi = self‐help CBTi, SH = sleep hygiene, stepcare = stepped‐care CBTi, TAU = treatment as usual, tCBTi = teleCBTi and TST = total sleep time.

We evaluated four secondary outcomes: SOL, WASO, TST and SE, with 15, 15, 15, and 17 studies included for each outcome, respectively. In comparison with TAU, dCBTi demonstrated greatest reduction in subjective SOL, with a mean decrease of 30.95 minutes (95% CI: −42.00 to −19.91), followed by gCBTi (−11.60; −18.12 to −5.08) and sCBTi (−10.83; −16.85 to −4.80). dCBTi showed significantly greater decrease in SOL compared to other regimens (Supporting Information S1: Material 8). The P‐score ranking indicated that dCBT‐I had the highest probability of being ranked first effective intervention for improving SOL (P‐score = 0.998; Supporting Information S1: Material 7).

Both dCBTi and gCBTi demonstrated significantly lower WASO scores compared to TAU (dCBTi: −25.62; −45.08 to −6.15; gCBTi: −22.03, −36.57 to −7.48; Supporting Information S1: Material 8). dCBT‐I and gCBT‐I showed the highest probabilities of being ranked as the leading delivery formats (P‐scores = 0.800 and 0.745, respectively; Supporting Information S1: Material 7).

Compared with TAU, dCBTi and sCBTi showed significant increase in TST compared to TAU (31.67 and 19.12 minutes), respectively (Supporting Information S1: Material 8). The P‐score suggested that dCBT‐I had a 77.2% probability of being the top‐ranked intervention (Supporting Information S1: Material 7).

Compared with TAU, dCBTi, sCBTi and gCBTi showed significant improvement in SE (14.63, 8.45, 8.35, respectively; Supporting Information S1: Material 8). Furthermore, dCBTi demonstrated significant different compared to sCBTi. The P‐score indicated that dCBT‐I was the most likely to be ranked first (0.904; Supporting Information S1: Material 7).

### 3.5 Heterogeneity and Inconsistency

No significant between‐study heterogeneity was detected for ISI, SOL, TST, and SE. In contrast, significant heterogeneity was observed for WASO (*Q* = 21.10, *p* = 0.0003, I^2^ = 80%; Supporting Information S1: Material 9). The global inconsistency testing indicated statistically significant inconsistency in the treatment effect of dCBTi on TST (*p* = 0.03) and SE (*p* = 0.001; Supporting Information S1: Material 10). While local inconsistency (node‐splitting analysis) did not identify any significant inconsistency for sleep outcomes, except for SE and SOL in the comparison between dCBTi and TAU and SOL in the comparison between dCBTi and sCBTi (*p* < 0.05; Supporting Information S1: Material 11).

### 3.6 Meta‐Regression Analysis

Cancer type (breast vs. non‐breast), treatment phase (active vs. post‐treatment), and the inclusion of insomnia criteria (broad definition vs. rigor definition) were entered as moderators in the metaregression analysis. Breast cancer diagnosis was the only significant moderator, influencing the effects of dCBTi on SOL (coefficient = 25.20, *p* = 0.015) and SE (coefficient = −16.17, *p* = 0.007; Supporting Information S1: Material 12). No other moderators reached statistical significance.

### 3.7 Sensitivity Analysis

A sensitivity analysis was conducted by excluding the study that required standard deviation imputation [45]. The results remained consistent with the primary analysis, confirming the robustness of our findings (Supporting Information S1: Material 13).

### 3.8 Publication Bias

Egger's regression test showed no evidence of publication bias for ISI, TST, and SE, whereas SOL and WASO showed potential small‐study effects (Supporting Information S1: Material 14).

### 3.9 Certainty of Evidence

The proportion of direct comparisons with low or very low confidence in the evidence was 69% for ISI score, 17% for SOL, 17% for WASO, 25% for TST, and 79% for SE (Supporting Information S1: Materials 15‐19). The primary concerns driving the downgrading of evidence quality were imprecision, which primarily stemmed from small sample sizes in single available trials, and incoherence between direct and indirect estimates within the network.

## 4 Discussion

In our NMA, several CBTi delivery modalities, including dCBTi, sCBTi, gCBTi, were considered among the most effective interventions for alleviating insomnia compared with other treatment regimens. This finding contrasts with Gao et al. (2023), reported larger effects for therapist‐guided interventions than for self‐guided digital programs [23]. Several methodological differences may account for the discrepancy. First, Gao et al. (2023) combined sCBTi and interactive dCBTi into a single intervention category, potentially obscuring important differences in efficacy between these delivery formats and influencing the pooled estimates [57]. Second, the larger number of studies included in our review yielded a different overall sample size and distribution, which may have contributed to the discrepancies in relative effect estimates [58]. Compared with previous reviews, our NMA incorporated a larger evidence base and evaluated CBTi according to distinct delivery modalities, providing a more comprehensive comparison of available treatment approaches. Therefore, our findings may provide a more robust estimate of the comparative effectiveness of different CBTi delivery modalities.

We found that dCBTi has substantial potential for relieving insomnia among individuals with cancers. In oncology populations, concomitant pain, fatigue, chemotherapy‐related side effects, and emotional distress, such as anxiety and depression, frequently disrupt sleep maintenance and contribute to insomnia [59, 60, 61, 62]. At the same time, these symptoms burdens and treatment demands may create substantial logistical barriers that limit patients' ability to attend traditional face‐to‐face sessions. In this context, dCBTi may offer a flexible and accessible alternative that allows patients to engage in treatment remotely and at their own pace. Importantly, digital health interventions have gained increasing acceptance in recent years, particularly following the COVID‐19 pandemic, with patients demonstrating increasing adaptability, willingness, and familiarity to engage with technology‐based healthcare services [63]. Nevertheless, the interpretation of these findings should be approached with caution, as the low certainty of evidence and the presence of network inconsistency related to dCBTi within the NMA may have influenced the robustness of the estimated treatment effects.

Across the secondary outcomes, dCBTi demonstrated significant improvements related to TAU. However, no significant differences were observed among most CBTi delivery formats, except for SOL. These findings may be interpreted within the framework of Espie's stepped care model for insomnia, which advocates the use of low‐intensity interventions as the foundation of insomnia care and reserves more intensive treatments for individuals who do not respond adequately [64]. Notably, our findings did not demonstrate a clear advantage of higher‐intensity delivery formats, such as individual face‐to‐face CBTi, over remotely delivered approaches. One possible explanation is that cancer survivors often face treatment‐related symptom burdens and practical barriers that may reduce engagement with traditional in‐person interventions. In contrast, digital and self‐guided modalities offer greater flexibility and accessibility, which may facilitate treatment adherence and help maintain therapeutic effectiveness [63].

The significant global inconsistency observed for dCBTi in TST and SE, together with the localized inconsistency identified for SE and SOL in the dCBTi versus TAU comparison and SOL in the dCBTi versus sCBTi comparison, suggests that treatment effects involving dCBTi may have been influenced by clinical heterogeneity. For the dCBTi versus sCBTi comparison, only one trial (Starling, 2024) provided direct evidence, and its participants were older and have more advanced cancer than those contributing to the indirect evidence. Age‐related decline in circadian regulation [65] and physiology burden associated with advanced cancer [66] may have attenuated responsiveness to digital behavioral interventions, potentially contributing to the discrepancy between direct and indirect estimates. Likewise, participants in the direct dCBTi versus TAU trial (Clara, 2025) were younger than those included in the indirect comparisons, and greater digital literacy and cognitive reserve among younger individuals may have enhanced engagement with dCBTi [67]. Although these observations provide plausible clinical explanations for the observed inconsistencies, they are based on post hoc examination of a limited number of studies and should therefore be considered hypothesis‐generating rather than confirmatory. Consequently, caution is warranted when interpreting and generalizing the pooled network estimates involving dCBTi across diverse oncology populations.

Notably, meta‐regression analyses identified breast cancer diagnosis as a significant moderator of dCBTi effects on both SOL and SE. One possible explanation is that breast cancer survivors frequently experience treatment‐related symptoms, such as vasomotor disturbances, endocrine therapy–related adverse effects, and persistent psychological distress, which may contribute to sleep disruption through mechanisms that are not fully addressed by behavioral interventions alone [68]. In addition, the included dCBTi interventions varied in treatment intensity, therapist involvement, interaction design, and cacner stage. Previous studies have suggested that therapist support and treatment engagement may influence adherence and treatment outcomes in digital behavioral interventions [10]. Moreover, Past research has shown that more advanced or active diseases can exacerbate pain, fatigue, nausea, steroid use, and irregular treatment schedules, all of which make insomnia difficult to disentangle from medical symptoms [69]. However, this specific finding must be interpreted with extreme caution. Within the dCBTi subset, the distribution of studies is highly limited and uneven, consisting of only one trial focusing exclusively on breast cancer and two trials on mixed cancer populations. Given this small sample size (*n* = 3) the observed interaction effect carries a higher risk of Type I error and may lack statistical robustness or generalizability. Moreover, the impact of these factors could not be formally examined because intervention characteristics were inconsistently reported across studies.

The Egger test was significant in SOL and WASO. This could result in an overestimation of the pooled treatment effects. Possible sources include selective publication of positive results and small sample sizes [70]. While a random‐effects model was used to mitigate between‐study variability, these findings highlight the need for cautious interpretation of effect sizes and underscore the importance of future large‐scale, high‐quality studies.

### 4.1 Limitation

Several limitations of this study should be stated. First, only seven of the 22 included studies (31.8%) were rated as having a low risk of bias, which may have compromised the internal validity of the network estimates. Second, although this NMA synthesized both direct and indirect evidence, some treatment comparisons were informed by a limited number of studies and participants, potentially reducing statistical power and increasing uncertainty around the effect estimates. In addition, multiple exploratory meta‐regression analyses were conducted without formal adjustment for multiple comparisons. Given the relatively small number of included studies, these analyses are susceptible to Type I error, and the identified moderator effects should therefore be regarded as hypothesis‐generating rather than confirmatory. Third, significant inconsistency was identified in selected network comparisons involving dCBTi. Although meta‐regression analyses suggested that breast cancer diagnosis may partially explain the inconsistency, the exploratory nature of these analyses and the limited, uneven distribution of available studies preclude definitive conclusions and limit the robustness and generalizability of the observed interaction effects. In addition, certain cancer types, such as head and neck cancers, may uniquely influence sleep outcomes because of disease‐ and treatment‐related effects on breathing patterns and sleep disturbances [71]. However, because most studies enrolled mixed cancer types, it was not possible to isolate the impact of specific cancer diagnoses on sleep outcomes. Finally, the included CBTi delivery modalities differed substantially in treatment intensity, therapist involvement, interaction design, and cancer stage. However, quantitative exploration of these factors was not feasible because of inconsistently reports across studies. Most trials described intervention intensity using broad ranges of session duration or program length rather than actual delivered treatment doses, and information regarding therapist support, adherence monitoring, and participant engagement was often limited.

### 4.2 Clinical Implications

Our findings suggested that dCBTi, sCBTi, and tCBTi may represent feasible and scalable options for managing insomnia in oncology populations, particularly for those experiencing treatment‐related symptom burden or barriers to attending face‐to‐face sessions. Within a stepped‐care framework, lower‐intensity CBTi modalities, such as sCBTi, may serve as an appropriate first‐line approach, with escalation to more intensive interventions when clinically indicated.

## 5 Conclusion

dCBTi demonstrated significant improvements across all sleep outcomes compared with TAU and was associated with greater reductions in insomnia severity than most other CBTi delivery modalities. Together with the favorable effects observed for gCBTi, sCBTi, and tCBTi, these findings suggest that remotely delivered CBTi may represent an accessible and scalable treatment option for cancer‐related insomnia. Nevertheless, the low to very low certainty of evidence and the inconsistency observed in selected network comparisons warrant cautious interpretation. Future high‐quality head‐to‐head trials are needed to establish the comparative effectiveness of different CBTi delivery modalities in oncology populations.

## Author Contributions

Conceptualization: Chen‐I Lee, Hsiao‐Yean Chiu. Methodology: Chen‐I Lee, Hsiao‐Yean Chiu. Investigation: Chen‐I Lee. Data curation: Chen‐I Lee, Tzu‐Hao Wang, Tzu‐Hao Wang. Formal analysis: Chen‐I Lee. Writing – original draft: Chen‐I Lee. Writing – review and editing: Chen‐I Lee, Hsiao‐Yean Chiu, Ya‐Wen Jan, Tsai‐Wei Huang. Supervision: Hsiao‐Yean Chiu. All authors have read and approved the final manuscript.

## Funding

This study was supported by the National Science and Technology Council of Taiwan (grant nos. NSTC 113‐2628‐B‐038‐003‐MY3 and NSTC 114‐2314‐B‐038‐094‐MY3).

## Conflicts of Interest

The authors declare no conflicts of interest.

## Supporting information


Supporting Information S1

## Data Availability

The data that support the findings of this study are available from the corresponding author upon reasonable request.
