# Supplementary material for: Comparative Effects of Various Delivery Methods of Cognitive Behavioral Therapy for Insomnia Among Patients With Cancers: Systematic Review and Network Meta‐Analysis
Source: Psychooncology. 2026 Aug 31;35(9):e70593. doi: 10.1002/pon.70593 (PMC13527890; doi:10.1002/pon.70593)
Supplement: Supplementary file 1 [file PON-35-e70593-s001.docx]

**Supplementary Online Material**

Supplementary Material 1 PRISMA NMA Checklist

Supplementary Material 2 Search strategy

Supplementary Material 3 Reason for excluded articles

Supplementary Material 4 Characteristic of included articles

Supplementary Material 5 The results of ROB 2.0

Supplementary Material 6 Network plots of secondary outcomes

Supplementary Material 7 The results of P-score according to different sleep outcomes

Supplementary Material 8 League tables of secondary outcomes

Supplementary Material 9 Heterogeneity of NMA

Supplementary Material 10 Results of global inconsistency in each sleep outcome

Supplementary Material 11 Results of local inconsistency in each sleep outcome

Supplementary Material 12 Meta-regression analysis

Supplementary Material 13 Sensitivity analysis for ISI The results of

Supplementary Material 14 Egger’s test for sleep outcomes

Supplementary Material 15 Certainty of evidence evaluating by CINeMA for ISI

Supplementary Material 16 Certainty of evidence evaluating by CINeMA for SOL

Supplementary Material 17 Certainty of evidence evaluating by CINeMA for WASO

Supplementary Material 18 Certainty of evidence evaluating by CINeMA for TST

Supplementary Material 19 Certainty of evidence evaluating by CINeMA for SE

Supplementary Material 20 The results of forest plots for sleep outcomes

Supplementary Material 21 Included articles

**Supplementary Material 1** PRISMA NMA Checklist of Items to Include When Reporting A Systematic Review Involving a Network Meta-analysis

| **Section/Topic** | **Item #** | **Checklist Item** | Reported on Page # |
| --- | --- | --- | --- |
| **TITLE** |  |  |  |
| Title | 1 | Identify the report as a systematic review *incorporating a network meta-analysis (or related form of meta-analysis).* | 1 |
|  |  |  |  |
| **ABSTRACT** |  |  | 3 |
| Structured summary | 2 | Provide a structured summary including, as applicable:  **Background:** main objectives  **Methods:** data sources; study eligibility criteria, participants, and interventions; study appraisal; and *synthesis methods, such as network meta-analysis.*  **Results:** number of studies and participants identified; summary estimates with corresponding confidence/credible intervals; *treatment rankings may also be discussed. Authors may choose to summarize pairwise comparisons against a chosen treatment included in their analyses for brevity.*  **Discussion/Conclusions:** limitations; conclusions and implications of findings.  **Other:** primary source of funding; systematic review registration number with registry name. |  |
|  |  |  |  |
| **INTRODUCTION** |  |  |  |
| Rationale | 3 | Describe the rationale for the review in the context of what is already known*, including mention of why a network meta-analysis has been conducted.* | 5 |
| Objectives | 4 | Provide an explicit statement of questions being addressed, with reference to participants, interventions, comparisons, outcomes, and study design (PICOS). | 6 |
|  |  |  |  |
| **METHODS** |  |  |  |
| Protocol and registration | 5 | Indicate whether a review protocol exists and if and where it can be accessed (e.g., Web address); and, if available, provide registration information, including registration number. | 6 |
| Eligibility criteria | 6 | Specify study characteristics (e.g., PICOS, length of follow-up) and report characteristics (e.g., years considered, language, publication status) used as criteria for eligibility, giving rationale. *Clearly describe eligible treatments included in the treatment network, and note whether any have been clustered or merged into the same node (with justification).* | 6, 7 |
| Information sources | 7 | Describe all information sources (e.g., databases with dates of coverage, contact with study authors to identify additional studies) in the search and date last searched. | 6 |
| Search | 8 | Present full electronic search strategy for at least one database, including any limits used, such that it could be repeated. | 6 |
| Study selection | 9 | State the process for selecting studies (i.e., screening, eligibility, included in systematic review, and, if applicable, included in the meta-analysis). | 7 |
| Data collection process | 10 | Describe method of data extraction from reports (e.g., piloted forms, independently, in duplicate) and any processes for obtaining and confirming data from investigators. | 7 |
| Data items | 11 | List and define all variables for which data were sought (e.g., PICOS, funding sources) and any assumptions and simplifications made. | 7 |
| **Geometry of the network** | **S1** | Describe methods used to explore the geometry of the treatment network under study and potential biases related to it. This should include how the evidence base has been graphically summarized for presentation, and what characteristics were compiled and used to describe the evidence base to readers. | - |
| Risk of bias within individual studies | 12 | Describe methods used for assessing risk of bias of individual studies (including specification of whether this was done at the study or outcome level), and how this information is to be used in any data synthesis. | 8 |
| Summary measures | 13 | State the principal summary measures (e.g., risk ratio, difference in means). *Also describe the use of additional summary measures assessed, such as treatment rankings and surface under the cumulative ranking curve (SUCRA) values, as well as modified approaches used to present summary findings from meta-analyses.* | 8 |
| Planned methods of analysis | 14 | Describe the methods of handling data and combining results of studies for each network meta-analysis. This should include, but not be limited to:   - *Handling of multi-arm trials;* - *Selection of variance structure;* - *Selection of prior distributions in Bayesian analyses; and* - *Assessment of model fit.* | 8, 9 |
| **Assessment of Inconsistency** | **S2** | Describe the statistical methods used to evaluate the agreement of direct and indirect evidence in the treatment network(s) studied. Describe efforts taken to address its presence when found. | 9 |
| Risk of bias across studies | 15 | Specify any assessment of risk of bias that may affect the cumulative evidence (e.g., publication bias, selective reporting within studies). | 8 |
| Additional analyses | 16 | Describe methods of additional analyses if done, indicating which were pre-specified. This may include, but not be limited to, the following:   - Sensitivity or subgroup analyses; - Meta-regression analyses; - *Alternative formulations of the treatment network; and* - *Use of alternative prior distributions for Bayesian analyses (if applicable).* | 9 |
|  |  |  |  |
| **RESULTS†** |  |  |  |
| Study selection | 17 | Give numbers of studies screened, assessed for eligibility, and included in the review, with reasons for exclusions at each stage, ideally with a flow diagram. | 10 |
| **Presentation of network structure** | **S3** | Provide a network graph of the included studies to enable visualization of the geometry of the treatment network. | Figure 2 |
| **Summary of network geometry** | **S4** | Provide a brief overview of characteristics of the treatment network. This may include commentary on the abundance of trials and randomized patients for the different interventions and pairwise comparisons in the network, gaps of evidence in the treatment network, and potential biases reflected by the network structure. | 10, 11, table 1 |
| Study characteristics | 18 | For each study, present characteristics for which data were extracted (e.g., study size, PICOS, follow-up period) and provide the citations. | 10, 11, S4 |
| Risk of bias within studies | 19 | Present data on risk of bias of each study and, if available, any outcome level assessment. | 11, S5 |
| Results of individual studies | 20 | For all outcomes considered (benefits or harms), present, for each study: 1) simple summary data for each intervention group, and 2) effect estimates and confidence intervals. *Modified approaches may be needed to deal with information from larger networks.* | Table 1 |
| Synthesis of results | 21 | Present results of each meta-analysis done, including confidence/credible intervals. *In larger networks, authors may focus on comparisons versus a particular comparator (e.g. placebo or standard care), with full findings presented in an appendix. League tables and forest plots may be considered to summarize pairwise comparisons.* If additional summary measures were explored (such as treatment rankings), these should also be presented. | 12, 13, Table 3, S7, S8 |
| **Exploration for inconsistency** | **S5** | Describe results from investigations of inconsistency. This may include such information as measures of model fit to compare consistency and inconsistency models, *P* values from statistical tests, or summary of inconsistency estimates from different parts of the treatment network. | 13, S10, S11 |
| Risk of bias across studies | 22 | Present results of any assessment of risk of bias across studies for the evidence base being studied. | 11, S5 |
| Results of additional analyses | 23 | Give results of additional analyses, if done (e.g., sensitivity or subgroup analyses, meta-regression analyses*, alternative network geometries studied, alternative choice of prior distributions for Bayesian analyses,* and so forth). | 14, S13, S15-19 |
|  |  |  |  |
| **DISCUSSION** |  |  |  |
| Summary of evidence | 24 | Summarize the main findings, including the strength of evidence for each main outcome; consider their relevance to key groups (e.g., healthcare providers, users, and policy-makers). | 14-16 |
| Limitations | 25 | Discuss limitations at study and outcome level (e.g., risk of bias), and at review level (e.g., incomplete retrieval of identified research, reporting bias). *Comment on the validity of the assumptions, such as transitivity and consistency. Comment on any concerns regarding network geometry (e.g., avoidance of certain comparisons).* | 17 |
| Conclusions | 26 | Provide a general interpretation of the results in the context of other evidence, and implications for future research. | 18 |
|  |  |  |  |
| **FUNDING** |  |  |  |
| Funding | 27 | Describe sources of funding for the systematic review and other support (e.g., supply of data); role of funders for the systematic review. This should also include information regarding whether funding has been received from manufacturers of treatments in the network and/or whether some of the authors are content experts with professional conflicts of interest that could affect use of treatments in the network. | 1 |

PICOS = population, intervention, comparators, outcomes, study design.

* Text in italics indicateS wording specific to reporting of network meta-analyses that has been added to guidance from the PRISMA statement.

† Authors may wish to plan for use of appendices to present all relevant information in full detail for items in this section.

**Supplementary Material 2** Search strategy

| **Databases** | **Keywords** |
| --- | --- |
| The PubMed, Embase, CINAHL and | (Cancer OR neoplasms) AND (sleep OR insomnia OR "sleep disorder" OR "sleep disturbance" OR "Sleep Initiation and Maintenance Disorders") AND (computer* OR digital OR "telemedicine-delivered" OR online OR "internet-based" OR "internet-delivered" OR "guided internet" OR "web- based" OR "telephone-based" OR "telephone-delivered" OR mobile OR "mobile-application based" OR "video-based" OR video OR individual OR "self-administered" OR group* OR "group-delivered" ) AND ("cognitive behavio* therapy for insomnia" OR "CBT-I" OR "behavio* therapy") |
| Cochrane database | (Cancer OR neoplasm*) AND (sleep OR insomnia OR (sleep NEXT disorder*) OR (sleep NEXT disturb*) OR (sleep NEXT initiation NEXT maintenance NEXT disorder*)) AND (computer* OR digital OR telemedicine-delivered OR online OR internet-based OR internet-delivered OR guided internet OR web-based OR telephone-based OR telephone-delivered OR mobile OR mobile-application-based OR video-based OR video OR individual OR self-administered OR group* OR group-delivered) AND ((cognitive NEXT behavio* NEXT therapy NEXT insomnia) OR CBT-I OR behavio* therapy) |

**Supplementary Material 3** Reason for excluded articles

| Excluded reasons | Titles of article |
| --- | --- |
| Not a RCT | 1. A step in the right direction: Making cognitive-behavioral therapy for insomnia more accessible to people diagnosed with cancer 2. Acupuncture versus cognitive behavioral therapy for pain among cancer survivors with insomnia: an exploratory analysis of a randomized clinical trial |
| Same study participants | 1. A danish language internet-delivered intervention for insomnia in cancer survivors: Effects on cancer-related fatigue 2. Randomized trial of a cognitive-behavioral intervention for insomnia in breast cancer survivors 3. The Comparative Impact of Mindfulness-Based Cancer Recovery (MBCR) and Cognitive Behavior Therapy for Insomnia (CBT-I) on Sleep and Mindfulness in Cancer Patients 4. Cognitive behavioral therapy for insomnia, but not armodafinil, improves fatigue in cancer survivors with insomnia: a randomized placebo-controlled trial 5. Acupuncture versus cognitive behavioral therapy for anxiety among cancer survivors with insomnia: an exploratory analysis of a randomized clinical trial 6. Addressing cancer-related fatigue through sleep: a secondary analysis of a randomized trial comparing acupuncture and cognitive behavioral therapy for insomnia 7. Efficacy of a stepped care approach to offer cognitive-behavioral therapy for insomnia in cancer patients 8. Video cognitive-behavioral therapy for insomnia in cancer patients: A cost-effective alternative 9. Is a video-based cognitive-behavioral therapy as efficacious as a professionally administered treatment for insomnia comorbid with cancer? preliminary results of a randomized controlled trial |
| Combined intervention | 1. Sleep Hygiene Education, ReadiWatch™ Actigraphy, and Telehealth Cognitive Behavioral Training for Insomnia for People With Prostate Cancer 2. Cognitive Behavioral Therapy for Insomnia Reduces Depression in Cancer Survivors 3. Randomized placebo-controlled trial of cognitive behavioral therapy and armodafinil for insomnia after cancer treatment |

**Supplementary Material 4** Characteristic of included articles

|  | Characteristic of included articles(n=22) | |
| --- | --- | --- |
| Age(mean) | 56.1 |  |
| Female, n (%) | 1805 | (88.5) |
| **Type of cancer** | |  |
| Breast cancer, n (%) | 10 | (45.4) |
| Mixed cancer, n (%) | 12 | (54.6) |
| **Type of CBT-I** | |  |
| Individual CBTi, n (%) | 8 | (36.4) |
| Group CBTi, n (%) | 5 | (22.7) |
| Digital CBTi, n (%) | 3 | (13.6) |
| Tele CBTi, n (%) | 2 | (9.1) |
| Self-help CBTi, n (%) | 5 | (22.7) |
| Step-care CBTi, n (%) | 1 | (4.5) |
| **Measurement** | |  |
| ISI, n (%) | 20 | (90.9) |
| SOL, n (%) | 17 | (77.3) |
| WASO, n (%) | 17 | (77.3) |
| TST, n (%) | 17 | (77.3) |
| SE, n (%) | 18 | (81.8) |

Abbreviations: BT = behavioral therapy, dCBTi = digital CBTi, EX = exercise, gCBTi = group CBTi, iCBTi = individual CBTi, ISI = insomnia sleep index, MBSR = mindfulness-based stress reduction, rTMS = repetitive transcranial magnetic stimulation, sCBTi = self-help CBTi, SE = sleep efficiency, SH = sleep hygiene, SOL = sleep onset latency, stepcare = stepped-care CBTi, TAU = treatment as usual, tCBTi = teleCBTi, TST = total sleep time, and WASO = wake after sleep onset

**Supplementary Material 5** The results of ROB 2.0

| Author(year) | Randomization process | Deviation from intended interventions | Missing outcome data | Measurement of the outcome | Selection of the reported results | overall |
| --- | --- | --- | --- | --- | --- | --- |
| Casault(2015) | LOW | LOW | SC | LOW | LOW | SC |
| Clara(2025) | LOW | SC | LOW | SC | LOW | SC |
| Dirksen(2008) | SC | HIGH | LOW | SC | LOW | HIGH |
| Espie(2008) | LOW | LOW | LOW | LOW | LOW | LOW |
| Fiorentino(2010) | LOW | HIGH | LOW | LOW | LOW | HIGH |
| Garland(2015) | LOW | LOW | LOW | LOW | LOW | LOW |
| Garland(2019) | LOW | LOW | LOW | LOW | LOW | LOW |
| Garland(2024) | SC | SC | LOW | LOW | LOW | SC |
| Hall(2022) | HIGH | SC | LOW | SC | LOW | HIGH |
| Irwin(2017) | LOW | LOW | LOW | LOW | LOW | LOW |
| Matthews(2014) | SC | LOW | SC | SC | LOW | SC |
| Mercier(2018) | LOW | LOW | LOW | LOW | LOW | LOW |
| Moon(2020) | LOW | SC | LOW | SC | SC | SC |
| Oswald(2022) | LOW | SC | LOW | SC | LOW | SC |
| Ritterband (2011) | LOW | LOW | LOW | SC | LOW | SC |
| Savard(2005) | SC | SC | LOW | LOW | LOW | SC |
| Savard(2014) | LOW | LOW | HIGH | LOW | LOW | HIGH |
| Savard (2021) | LOW | LOW | LOW | LOW | LOW | LOW |
| Starling(2024) | SC | SC | LOW | SC | LOW | SC |
| Tian(2025) | SC | SC | HIGH | SC | LOW | HIGH |
| Zachariae (2018) | LOW | LOW | LOW | LOW | LOW | LOW |
| Zheng(2015) | SC | LOW | LOW | SC | LOW | SC |

Abbreviation: LOW=low risk, SC=some concern, HIGH=high risk

**Supplementary Material 6** Network plots of secondary outcomes

(1) SOL	(2) WASO

(3) TST	(4) SE

Abbreviations: BT = behavioral therapy, dCBTi = digital CBTi, EX = exercise, gCBTi = group CBTi, iCBTi = individual CBTi, ISI = insomnia sleep index, MBSR = mindfulness-based stress reduction, rTMS = repetitive transcranial magnetic stimulation, sCBTi = self-help CBTi, SE = sleep efficiency, SH = sleep hygiene, SOL = sleep onset latency, stepcare = stepped-care CBTi, TAU = treatment as usual, tCBTi = teleCBTi, TST = total sleep time, and WASO = wake after sleep onset

**Supplementary Material 7** The results of P-score according to different sleep outcomes

(1) ISI	(2) SOL	(3) WASO

(4) TST	(5) SE

Abbreviations: BT = behavioral therapy, dCBTi = digital CBTi, EX = exercise, gCBTi = group CBTi, iCBTi = individual CBTi, ISI = insomnia sleep index, MBSR = mindfulness-based stress reduction, rTMS = repetitive transcranial magnetic stimulation, sCBTi = self-help CBTi, SE = sleep efficiency, SH = sleep hygiene, SOL = sleep onset latency, stepcare = stepped-care CBTi, TAU = treatment as usual, tCBTi = teleCBTi, TST = total sleep time, and WASO = wake after sleep onset

**Supplementary Material 8** League tables of secondary outcomes

(1) SOL

| dCBTi | . | -12.80  (-25.52 to -0.08) | . | . | . | -45.00  (-62.61 to -27.39) | . | . | . |
| --- | --- | --- | --- | --- | --- | --- | --- | --- | --- |
| **-19.36**  **(-31.99 to -6.72)** | gCBTi | . | . | . | -8.10  (-20.85 to 4.65) | -10.96  (-17.82 to -4.11) | . | -16.71  (-28.67 to -4.75) | . |
| **-20.13**  **(-30.64 to -9.61)** | -0.77  ( -9.22 to 7.68) | sCBTi | 1.90  (-11.40 to 15.20) | . | -1.80  (-17.53 to 13.93) | -9.46  (-16.02 to -2.90) | . | . | . |
| **-20.56**  **(-36.00 to -5.11)** | -1.20  (-14.49 to 12.09) | -0.43  (-12.34 to 11.49) | iCBTi | -2.10  (-13.37 to 9.17) | . | -8.60  (-21.18 to 3.98) | -12.76  (-29.34 to 3.82) | . | -16.33  (-29.02 to -3.64) |
| **-22.66**  **(-41.77 to -3.54)** | -3.30  (-20.72 to 4.12) | -2.53  (-18.93 to 13.87) | -2.10  (-13.37 to 9.17) | stepcare | . | . | . | . | . |
| **-25.26**  **(-40.14 to -10.39)** | -5.91  (-16.36 to 4.55) | -5.14  (-16.28 to 6.00) | -4.71  (-20.33 to 10.91) | -2.61  (-21.87 to 16.65) | EX | . | . | . | . |
| **-30.95**  **(-42.00 to -19.91)** | **-11.60**  **(-18.12 to -5.08)** | **-10.83**  **(-16.85 to -4.80)** | -10.40  (-22.12 to 1.32) | -8.30  (-24.56 to 7.96) | -5.69  (-16.69 to 5.31) | TAU | . | . | . |
| **-33.32**  **(-55.97 to -10.66)** | -13.96  (-35.20 to 7.29) | -13.19  (-33.61 to 7.23) | -12.76  (-29.34 to 3.82) | -10.66  (-30.71 to 9.39) | -8.05  (-30.83 to 14.72) | -2.36  (-22.66 to 17.94) | BT | . | . |
| **-36.07**  **(-53.46 to -18.67)** | **-16.71**  **(-28.67 to -4.75)** | **-15.94**  **(-30.58 to -1.30)** | -15.51  (-33.39 to 2.36) | -13.41  (-34.54 to 7.72) | -10.80  (-26.69 to 5.08) | -5.11  (-18.73 to 8.51) | -2.75  (-27.13 to 21.63) | MBSR | . |
| **-36.89**  **(-56.87 to -16.90)** | -17.53  (-35.90 to 0.84) | -16.76  (-34.17 to 0.65) | **-16.33**  **(-29.02 to -3.64)** | -14.23  (-31.20 to 2.74) | -11.62  (-31.74 to 8.50) | -5.93  (-23.20 to 11.34) | -3.57  (-24.45 to 17.31) | -0.82  (-22.74 to 21.10) | Acupuncture |

(2) WASO

| dCBTi | . | . | . | 0.30  (-25.59 to 26.19) | . | . | . | . | -39.00  (-65.41 to -12.59) |
| --- | --- | --- | --- | --- | --- | --- | --- | --- | --- |
| -3.59  (-27.47 to 20.29) | gCBTi | . | -8.20  (-33.67 to 17.27) | . | -12.17  (-38.03 to 13.69) | . | . | . | -22.33  (-37.91 to -6.76) |
| -7.92  (-38.27 to 22.42) | -4.34  (-32.66 to 23.99) | iCBTi | . | -4.40  (-32.26 to 23.46) | . | -8.26  (-36.54 to 20.02) | -8.94  (-34.48 to 16.60) | -8.90  (-33.68 to 15.88) | -17.89  (-44.74 to 8.96) |
| -12.60  (-40.46 to 15.26) | -9.01  (-29.67 to 11.64) | -4.67  (-36.51 to 27.16) | EX | 1.10  (-28.01 to 30.21) | . | . | . | . | . |
| -12.56  (-31.95 to 6.83) | -8.97  (-26.71 to 8.77) | -4.63  (-29.55 to 20.28) | 0.04  (-21.60 to 21.68) | sCBTi | . | . | . | . | -9.47  (-22.72 to 3.79) |
| -15.76  (-50.95 to 19.44) | -12.17  (-38.03 to 13.69) | -7.83  (-46.18 to 30.52) | -3.16  (-36.25 to 29.93) | -3.20  (-34.56 to 28.15) | MBSR | . | . | . | . |
| -16.18  (-57.67 to 25.30) | -12.60  (-52.62 to 27.43) | -8.26  (-36.54 to 20.02) | -3.59  (-46.17 to 39.00) | -3.63  (-41.32 to 34.06) | -0.43  (-48.08 to 47.23) | BT | . | . | . |
| -16.86  (-56.53 to 22.80) | -13.28  (-51.41 to 24.86) | -8.94  (-34.48 to 16.60) | -4.27  (-45.08 to 36.55) | -4.31  (-39.98 to 31.37) | -1.11  (-47.18 to 44.97) | -0.68  (-38.79 to 37.43) | Acupuncture | . | . |
| -16.82  (-56.00 to 22.35) | -13.24  (-50.87 to 24.39) | -8.90  (-33.68 to 15.88) | -4.23  (-44.57 to 36.12) | -4.27  (-39.40 to 30.87) | -1.07  (-46.72 to 44.59) | -0.64  (-38.24 to 36.96) | 0.04  (-35.54 to 35.62) | stepcare | . |
| **-25.62**  **(-45.08 to -6.15)** | **-22.03**  **(-36.57 to -7.48)** | -17.69  (-42.37 to 6.98) | -13.02  (-34.74 to 8.71) | **-13.06**  **(-25.01 to -1.10)** | -9.86  (-39.52 to 19.81) | -9.43  (-46.97 to 28.10) | -8.75  (-44.26 to 26.76) | -8.79  (-43.76 to 26.18) | TAU |

(3) TST

| dCBTi | . | . | . | -10.80  (-47.49 to 25.89) | . | . | . | . | 51.00  ( 17.63 to 84.37) |
| --- | --- | --- | --- | --- | --- | --- | --- | --- | --- |
| -0.05  (-45.60 to 45.51) | BT | . | . | . | . | . | . | 31.33  ( -0.49 to 63.15) | . |
| 4.48  (-40.85 to 49.82) | 4.53  (-40.24 to 49.30) | Acupuncture | . | . | . | . | . | 26.80  ( -4.70 to 58.30) | . |
| 10.58  (-33.25 to 54.41) | 10.63  (-32.62 to 53.88) | 6.10  (-36.91 to 49.11) | stepcare | . | . | . | . | 20.70  ( -8.59 to 49.99) | . |
| 12.55  (-13.50 to 38.61) | 12.60  (-26.42 to 51.62) | 8.07  (-30.69 to 46.83) | 1.97  (-35.03 to 38.96) | sCBTi | -14.20  (-56.62 to 28.22) | . | . | 11.20  (-14.22 to 36.62) | 16.58  ( -0.08 to 33.24) |
| 13.12  (-24.95 to 51.19) | 13.17  (-34.85 to 61.18) | 8.64  (-39.17 to 56.44) | 2.54  (-43.85 to 48.92) | 0.57  (-29.54 to 30.67) | EX | -8.00  (-41.95 to 25.95) | . | . | . |
| 14.58  (-17.11 to 46.27) | 14.63  (-28.60 to 57.85) | 10.10  (-32.89 to 53.08) | 4.00  (-37.41 to 45.40) | 2.03  (-21.38 to 25.43) | 1.46  (-26.58 to 29.50) | gCBTi | 6.60  (-21.97 to 35.17) | . | 13.62  ( -6.94 to 34.19) |
| 21.18  (-21.49 to 63.85) | 21.23  (-30.59 to 73.04) | 16.70  (-34.92 to 68.31) | 10.60  (-39.71 to 60.90) | 8.63  (-28.31 to 45.56) | 8.06  (-31.97 to 48.09) | 6.60  (-21.97 to 35.17) | MBSR | . | . |
| 31.28  ( -1.32 to 63.89) | 31.33  ( -0.49 to 63.15) | 26.80  ( -4.70 to 58.30) | 20.70  ( -8.59 to 49.99) | 18.73  ( -3.86 to 41.33) | 18.16  (-17.80 to 54.13) | 16.70  (-12.55 to 45.96) | 10.10  (-30.79 to 51.00) | iCBTi | -7.20  (-32.68 to 18.28) |
| **31.67**  **( 6.04 to 57.31)** | 31.72  ( -7.31 to 70.76) | 27.19  (-11.58 to 65.97) | 21.09  (-15.92 to 58.10) | **19.12**  **( 3.88 to 34.36)** | 18.56  (-11.29 to 48.40) | 17.10  ( -2.23 to 36.43) | 10.50  (-24.00 to 44.99) | 0.39  (-22.22 to 23.01) | TAU |

(4) SE

| dCBTi | . | 2.00  ( -4.80 to 8.80) | . | . | . | . | . | . | . | . | 21.30  ( 12.72 to 29.88) |
| --- | --- | --- | --- | --- | --- | --- | --- | --- | --- | --- | --- |
| 1.63  (-13.53 to 16.80) | tCBTi | . | . | . | . | 5.58  ( -3.49 to 14.65) | . | . | . | . | 13.00  ( -1.01 to 27.01) |
| **6.18**  **(0.66 to 11.70)** | 4.55  ( -9.95 to 19.05) | sCBTi | . | -2.00  (-11.21 to 7.21) | . | . | . | . | 1.50  ( -7.73 to 10.73) | . | 7.37  ( 3.20 to 11.54) |
| 6.29  ( -0.84 to 13.41) | 4.65  (-10.03 to 19.34) | 0.10  ( -5.34 to 5.55) | gCBTi | . | . | . | . | . | 5.60  ( -2.37 to 13.57) | 7.91  ( 0.41 to 15.41) | 7.73  ( 3.05 to 12.41) |
| 6.59  ( -2.98 to 16.17) | 4.96  (-11.21 to 21.13) | 0.41  ( -7.80 to 8.62) | 0.31  ( -8.80 to 9.42) | iCBTi | 0.05  ( -6.75 to 6.85) | . | 2.30  ( -5.00 to 9.60) | 3.40  ( -5.08 to 11.88) | . | . | 6.30  ( -2.32 to 14.92) |
| 6.64  ( -5.10 to 18.38) | 5.01  (-12.53 to 22.55) | 0.46  (-10.20 to 11.12) | 0.36  (-11.01 to 11.72) | 0.05  ( -6.75 to 6.85) | Acupuncture | . | . | . | . | . | . |
| 7.21  (-10.46 to 24.88) | 5.58  ( -3.49 to 14.65) | 1.03  (-16.07 to 18.13) | 0.93  (-16.33 to 18.19) | 0.62  (-17.92 to 19.16) | 0.57  (-19.18 to 20.32) | SH | . | . | . | . | . |
| 8.89  ( -3.15 to 20.93) | 7.26  (-10.48 to 25.00) | 2.71  ( -8.28 to 13.70) | 2.61  ( -9.07 to 14.28) | 2.30  ( -5.00 to 9.60) | 2.25  ( -7.72 to 12.22) | 1.68  (-18.25 to 21.60) | stepcare | . | . | . | . |
| 9.99  ( -2.80 to 22.78) | 8.36  ( -9.90 to 26.62) | 3.81  ( -7.99 to 15.62) | 3.71  ( -8.74 to 16.15) | 3.40  ( -5.08 to 11.88) | 3.35  ( -7.52 to 14.22) | 2.78  (-17.61 to 23.17) | 1.10  (-10.09 to 12.29) | BT | . | . | . |
| **10.09**  **( 1.65 to 18.53)** | 8.46  ( -7.11 to 24.03) | 3.91  ( -2.88 to 10.70) | 3.80  ( -2.66 to 10.27) | 3.50  ( -6.77 to 13.77) | 3.45  ( -8.87 to 15.76) | 2.88  (-15.14 to 20.90) | 1.20  (-11.40 to 13.80) | 0.10  (-13.22 to 13.42) | EX | . | . |
| **14.20**  **( 3.85 to 24.54)** | 12.56  ( -3.92 to 29.05) | 8.01  ( -1.25 to 17.28) | **7.91**  **( 0.41 to 15.41)** | 7.60  ( -4.20 to 19.40) | 7.55  ( -6.06 to 21.17) | 6.98  (-11.83 to 25.80) | 5.30  ( -8.57 to 19.18) | 4.20  (-10.33 to 18.73) | 4.11  ( -5.79 to 14.01) | MBSR | . |
| **14.63**  **( 8.83 to 20.43)** | 13.00  ( -1.01 to 27.01) | **8.45**  **( 4.72 to 12.19)** | **8.35**  **( 3.95 to 12.74)** | 8.04  ( -0.03 to 16.11) | 7.99  ( -2.56 to 18.54) | 7.42  ( -9.27 to 24.11) | 5.74  ( -5.14 to 16.62) | 4.64  ( -7.07 to 16.35) | 4.54  ( -2.25 to 11.34) | 0.44  ( -8.25 to 9.13) | TAU |

Values in bold indicate significant.

Abbreviations: BT = behavioral therapy, dCBTi = digital CBTi, EX = exercise, gCBTi = group CBTi, iCBTi = individual CBTi, ISI = insomnia sleep index, MBSR = mindfulness-based stress reduction, rTMS = repetitive transcranial magnetic stimulation, sCBTi = self-help CBTi, SE = sleep efficiency, SH = sleep hygiene, SOL = sleep onset latency, stepcare = stepped-care CBTi, TAU = treatment as usual, tCBTi = teleCBTi, TST = total sleep time, and WASO = wake after sleep onset

**Supplementary Material 9** Heterogeneity of NMA

| Outcome | I2 | Q | d.f. | P value |
| --- | --- | --- | --- | --- |
| ISI | 0% | 4.28 | 4 | 0.369 |
| SOL | 59.1% | 8.36 | 4 | 0.079 |
| WASO | 80.6% | 21.1 | 4 | 0.0003 |
| TST | 38.5% | 2.72 | 4 | 0.606 |
| SE | 70.2% | 2.44 | 4 | 0.655 |

Abbreviations: ISI = insomnia sleep index, SE = sleep efficiency, SOL = sleep onset latency, TST = total sleep time, and WASO = wake after sleep onset

**Supplementary Material 10** Results of global inconsistency in each sleep outcome

| Outcomes | Chi^2^ | P value |
| --- | --- | --- |
| ISI | 2.77 | 0.597 |
| SOL | 5.09 | 0.165 |
| WASO | 1.96 | 0.581 |
| TST | 8.66 | 0.034 |
| SE | 21.08 | 0.0001 |

Abbreviations: ISI = insomnia sleep index, SE = sleep efficiency, SOL = sleep onset latency, TST = total sleep time, and WASO = wake after sleep onset

**Supplementary Material** **11** Results of local inconsistency in each sleep outcome

(1) ISI

(2) SOL

(3) WASO

(4) TST

(5) SE

Abbreviations: BT = behavioral therapy, dCBTi = digital CBTi, EX = exercise, gCBTi = group CBTi, iCBTi = individual CBTi, ISI = insomnia sleep index, MBSR = mindfulness-based stress reduction, rTMS = repetitive transcranial magnetic stimulation, sCBTi = self-help CBTi, SE = sleep efficiency, SH = sleep hygiene, SOL = sleep onset latency, stepcare = stepped-care CBTi, TAU = treatment as usual, tCBTi = teleCBTi, TST = total sleep time, and WASO = wake after sleep onset

**Supplementary Material 12** Meta-regression analysis

| **Insomnia severity index** | | |
| --- | --- | --- |
| **Intervention** | **Coefficient** | **P value** |
| Breast cancer | | |
| SH | 1.429644 | 0.532 |
| dCBTi | -1.348427 | 0.349 |
| gCBTi | -2.878493 | 0.983 |
| iCBTi | -4.076971 | 0.958 |
| sCBTi | 0.5109065 | 0.748 |
| tCBTi | 3.77981 | 0.075 |
|  | **Chi2** | **P value** |
| Overall effect | 4.45 | 0.6157 |
| Treatment stage |  |  |
| gCBTi | 1.12959 | 0.462 |
| sCBTi | -.8850265 | 0.618 |
|  | **Chi2** | **P value** |
| Overall effect | 0.79 | 0.6739 |
| Inclusion criteria of insomnia |  |  |
| dCBTi | 2.366989 | 0.493 |
| iCBTi | 1.101222 | 0.653 |
| tCBTi | -3.593602 | 0.088 |
|  | **Chi2** | **P value** |
| Overall effect | 3.63 | 0.3041 |
| **Sleep onset latency** |  |  |
| **Intervention** | **Coefficient** | **P value** |
| Breast cancer |  |  |
| Ex | 11.47571 | 0.303 |
| **dCBTi** | **25.20215** | **0.015** |
| gCBTi | 3.389899 | 0.623 |
| iCBTi | -5.118325 | 0.991 |
| sCBTi | 7.487604 | 0.222 |
|  | **Chi2** | **P value** |
| Overall effect | 7.32 | 0.1978 |
| Treatment stage |  |  |
| gCBTi | 8.938224 | 0.151 |
| sCBTi | -3.516025 | 0.663 |
|  | **Chi2** | **P value** |
| Overall effect | 2.32 | 0.3135 |
| Inclusion criteria of insomnia |  |  |
| EX | 6.114342 | 0.625 |
| iCBTi | 3.512854 | 0.995 |
| sCBTi | -4.251677 | 0.684 |
|  | **Chi2** | **P value** |
| Overall effect | 0.45 | 0.9288 |
| **Wake After Sleep Onset** |  |  |
| **Intervention** | **Coefficient** | **P value** |
| Breast cancer |  |  |
| Ex | -5.779846 | 0.851 |
| dCBTi | 29.70609 | 0.272 |
| gCBTi | 3.656536 | 0.868 |
| iCBTi | -10.68809 | 0.988 |
| sCBTi | 0.1286442 | 0.994 |
|  | **Chi2** | **P value** |
| Overall effect | 1.36 | 0.9283 |
| Treatment stage |  |  |
| gCBTi | 23.89717 | 0.088 |
| sCBTi | -8.904292 | 0.566 |
|  | **Chi2** | **P value** |
| Overall effect | 3.33 | 0.1888 |
| Inclusion criteria of insomnia |  |  |
| EX | 0.5984531 | 0.981 |
| iCBTi | 15.16828 | 0.984 |
| sCBTi | 17.53453 | 0.318 |
|  | **Chi2** | **P value** |
| Overall effect | 1.01 | 0.7987 |
| **Total sleep time** |  |  |
| **Intervention** | **Coefficient** | **P value** |
| Breast cancer |  |  |
| Ex | -34.52202 | 0.313 |
| dCBTi | -47.71907 | 0.078 |
| gCBTi | -10.57383 | 0.648 |
| iCBTi | 11.49314 | 0.994 |
| sCBTi | -8.866141 | 0.637 |
|  | **Chi2** | **P value** |
| Overall effect | 4.28 | 0.5104 |
| Treatment stage |  |  |
| gCBTi | 10.46722 | 0.659 |
| sCBTi | 4.38206 | 0.864 |
|  | **Chi2** | **P value** |
| Overall effect | 0.22 | 0.8961 |
| Inclusion criteria of insomnia |  |  |
| EX | -30.17875 | 0.366 |
| iCBTi | -15.79847 | 0.990 |
| sCBTi | 1.615425 | 0.952 |
|  | **Chi2** | **P value** |
| Overall effect | 0.84 | 0.8393 |
| **Sleep efficiency** |  |  |
| **Intervention** | **Coefficient** | **P value** |
| Breast cancer |  |  |
| EX | -3.030535 | 0.701 |
| **dCBTi** | **-16.16824** | **0.007** |
| gCBTi | 1.585813 | 0.761 |
| iCBTi | 6.58264 | 0.320 |
| sCBTi | -2.18886 | 0.601 |
| tCBTi | 10.21 | 0.973 |
|  | **Chi2** | **P value** |
| Overall effect | 8.94 | 0.1768 |
| Treatment stage |  |  |
| gCBTi | -1.700252 | 0.805 |
| sCBTi | 2.323184 | 0.726 |
|  | **Chi2** | **P value** |
| Overall effect | 0.19 | 0.9096 |
| Inclusion criteria of insomnia |  |  |
| EX | -3.278677 | 0.735 |
| iCBTi | -7.499875 | 0.380 |
| sCBTi | -1.049151 | 0.882 |
| tCBTi | -10.21 | 0.973 |
|  | **Chi2** | **P value** |
| Overall effect | 0.86 | 0.9295 |

Abbreviations: BT = behavioral therapy, dCBTi = digital CBTi, EX = exercise, gCBTi = group CBTi, iCBTi = individual CBTi, ISI = insomnia sleep index, MBSR = mindfulness-based stress reduction, rTMS = repetitive transcranial magnetic stimulation, sCBTi = self-help CBTi, SE = sleep efficiency, SH = sleep hygiene, SOL = sleep onset latency, stepcare = stepped-care CBTi, TAU = treatment as usual, tCBTi = teleCBTi, TST = total sleep time, and WASO = wake after sleep onset

**Supplementary Material 13** Sensitivity analysis for ISI (A) Network plot (B) p score (C) Forest plot (D) League table

(A) **(B)**

| **intervention** | **P score** |
| --- | --- |
| dCBTi | 0.9727 |
| tCBTi | 0.8998 |
| Herb | 0.8245 |
| sCBTi | 0.6760 |
| gCBTi | 0.6260 |
| iCBTi | 0.5787 |
| EX | 0.4201 |
| rtms | 0.3939 |
| stepcare | 0.3919 |
| SH | 0.3325 |
| Acupuncture | 0.2106 |
| MBSR | 0.1517 |
| TAU | 0.0214 |

**(C)**

**(D)**

| dCBTi | . | . | -5.30  ( -7.57 to -3.03) | . | . | . | . | . | . | . | . | -9.43  (-10.65 to -8.21) |
| --- | --- | --- | --- | --- | --- | --- | --- | --- | --- | --- | --- | --- |
| -0.97  ( -2.68 to 0.75) | tCBTi | . | . | . | . | . | . | . | -5.41  ( -8.18 to -2.65) | . | . | -8.83  (-10.21 to -7.44) |
| -2.09  ( -6.70 to 2.51) | -1.13  ( -5.70 to 3.44) | Herb | . | . | -3.00  ( -7.06 to 1.06) | . | . | . | . | . | . | . |
| -4.36  ( -5.68 to -3.04) | -3.39  ( -5.04 to -1.75) | -2.27  ( -6.84 to 2.31) | sCBTi | . | 2.00  ( -3.42 to 7.42) | -1.80  ( -5.99 to 2.39) | . | . | . | . | . | -5.56  ( -6.63 to -4.49) |
| -4.64  ( -5.88 to -3.40) | -3.68  ( -5.12 to -2.24) | -2.55  ( -7.06 to 1.96) | -0.28  ( -1.43 to 0.86) | gCBTi | . | . | . | . | . | . | -3.78  ( -5.38 to -2.18) | -5.06  ( -5.64 to -4.48) |
| -5.09  ( -7.26 to -2.93) | -4.13  ( -6.22 to -2.04) | -3.00  ( -7.06 to 1.06) | -0.73  ( -2.83 to 1.37) | -0.45  ( -2.41 to 1.51) | iCBTi | . | -1.30  ( -5.42 to 2.82) | -1.20  ( -2.60 to 0.20) | -1.93  ( -4.33 to 0.47) | -2.60  ( -4.09 to -1.11) | . | -4.28  ( -6.42 to -2.15) |
| -6.16  (-10.56 to -1.76) | -5.19  ( -9.70 to -0.69) | -4.07  (-10.27 to 2.14) | -1.80  ( -5.99 to 2.39) | -1.52  ( -5.86 to 2.83) | -1.07  ( -5.76 to 3.62) | EX | . | . | . | . | . | . |
| -6.39  (-11.05 to -1.74) | -5.43  (-10.05 to -0.81) | -4.30  (-10.09 to 1.49) | -2.03  ( -6.66 to 2.59) | -1.75  ( -6.31 to 2.82) | -1.30  ( -5.42 to 2.82) | -0.23  ( -6.48 to 6.01) | rtms | . | . | . | . | . |
| -6.29  ( -8.87 to -3.71) | -5.33  ( -7.84 to -2.81) | -4.20  ( -8.50 to 0.10) | -1.93  ( -4.46 to 0.59) | -1.65  ( -4.06 to 0.76) | -1.20  ( -2.60 to 0.20) | -0.13  ( -5.03 to 4.76) | 0.10  ( -4.25 to 4.45) | stepcare | . | . | . | . |
| -6.75  ( -9.23 to -4.27) | -5.78  ( -7.95 to -3.61) | -4.65  ( -9.19 to -0.12) | -2.39  ( -4.81 to 0.04) | -2.10  ( -4.40 to 0.20) | -1.65  ( -3.67 to 0.37) | -0.59  ( -5.43 to 4.26) | -0.35  ( -4.95 to 4.24) | -0.45  ( -2.91 to 2.00) | SH | . | . | . |
| -7.69  (-10.32 to -5.06) | -6.73  ( -9.29 to -4.16) | -5.60  ( -9.93 to -1.27) | -3.33  ( -5.91 to -0.76) | -3.05  ( -5.51 to -0.59) | -2.60  ( -4.09 to -1.11) | -1.53  ( -6.46 to 3.39) | -1.30  ( -5.68 to 3.08) | -1.40  ( -3.45 to 0.65) | -0.95  ( -3.46 to 1.57) | Acupuncture | . | . |
| -8.42  (-10.45 to -6.40) | -7.46  ( -9.61 to -5.31) | -6.33  (-11.12 to -1.55) | -4.06  ( -6.03 to -2.10) | -3.78  ( -5.38 to -2.18) | -3.33  ( -5.86 to -0.80) | -2.26  ( -6.90 to 2.37) | -2.03  ( -6.87 to 2.81) | -2.13  ( -5.02 to 0.76) | -1.68  ( -4.48 to 1.12) | -0.73  ( -3.67 to 2.20) | MBSR | . |
| -9.70  (-10.80 to -8.60) | -8.74  (-10.05 to -7.42) | -7.61  (-12.08 to -3.13) | -5.34  ( -6.33 to -4.35) | -5.06  ( -5.64 to -4.48) | -4.61  ( -6.48 to -2.74) | -3.54  ( -7.85 to 0.77) | -3.31  ( -7.84 to 1.22) | -3.41  ( -5.75 to -1.07) | -2.95  ( -5.18 to -0.73) | -2.01  ( -4.40 to 0.39) | -1.28  ( -2.98 to 0.42) | TAU |

Abbreviations: BT = behavioral therapy, dCBTi = digital CBTi, EX = exercise, gCBTi = group CBTi, iCBTi = individual CBTi, ISI = insomnia sleep index, MBSR = mindfulness-based stress reduction, rTMS = repetitive transcranial magnetic stimulation, sCBTi = self-help CBTi, SE = sleep efficiency, SH = sleep hygiene, SOL = sleep onset latency, stepcare = stepped-care CBTi, TAU = treatment as usual, tCBTi = teleCBTi, TST = total sleep time, and WASO = wake after sleep onset

**Supplementary Material 14** The results of Egger’s test for sleep outcomes

(1) ISI	(2) SOL

(3) WASO	(4) TST

(5) SE

Abbreviations: ISI = insomnia sleep index, SE = sleep efficiency, SOL = sleep onset latency, TST = total sleep time, and WASO = wake after sleep onset

**Supplementary Material 15** Certainty of evidence evaluating by CINeMA for ISI

| Comparison | Number of studies | Within-study bias | Reporting bias | Indirectness | Imprecision | Heterogeneity | Incoherence | Confidence rating | Reason(s) for downgrading |
| --- | --- | --- | --- | --- | --- | --- | --- | --- | --- |
| **Mixed evidence** | | | | | | | | | |
| Acupuncture:iCBTi | 1 | No concerns | Low risk | No concern | Major concerns | No concerns | Major concerns | Low | Imprecision, Incoherence |
| BT:iCBTi | 1 | Some concerns | Low risk | No concern | Major concerns | No concerns | Major concerns | Low | Imprecision, Incoherence |
| EX:sCBTi | 1 | No concerns | Low risk | No concern | Major concerns | No concerns | Major concerns | Low | Imprecision, Incoherence |
| Herb:iCBTi | 1 | Some concerns | Low risk | No concern | Major concerns | No concerns | Major concerns | Low | Imprecision, Incoherence |
| MBSR:gCBTi | 1 | No concerns | Low risk | No concern | Major concerns | No concerns | Major concerns | Low | Imprecision, Incoherence |
| SH:iCBTi | 1 | Major concerns | Low risk | No concern | Major concerns | No concerns | No concerns | Low | Within-study bias, Imprecision |
| SH:tCBTi | 1 | Major concerns | Low risk | No concern | Some concerns | Some concerns | No concerns | Low | Within-study bias, Imprecision, Heterogeneity |
| TAU:dCBTi | 2 | Some concerns | Low risk | No concern | No concerns | Some concerns | No concerns | Moderate | Within-study bias, Heterogeneity |
| TAU:gCBTi | 2 | Some concerns | Low risk | No concern | No concerns | No concerns | Major concerns | Moderate | Incoherence |
| TAU:iCBTi | 2 | Major concerns | Low risk | No concern | Some concerns | Some concerns | No concerns | Low | Within-study bias, Imprecision, Heterogeneity |
| TAU:sCBTi | 3 | Some concerns | Low risk | No concern | No concerns | Major concerns | No concerns | Moderate | Within-study bias, Heterogeneity |
| TAU:tCBTi | 2 | Some concerns | Low risk | No concern | No concerns | Some concerns | No concerns | Moderate | Within-study bias, Heterogeneity |
| dCBTi:sCBTi | 1 | Some concerns | Low risk | No concern | Some concerns | Some concerns | No concerns | Moderate | Imprecision, Heterogeneity |
| iCBTi:rtms | 1 | Major concerns | Low risk | No concern | Major concerns | No concerns | Major concerns | Very low | Within-study bias, Imprecision, Incoherence |
| iCBTi:sCBTi | 1 | Major concerns | Low risk | No concern | Major concerns | No concerns | No concerns | Low | Within-study bias, Imprecision |
| iCBTi:stepcare | 1 | No concerns | Low risk | No concern | Major concerns | No concerns | Major concerns | Low | Imprecision, Incoherence |
| **Indirect evidence** | | | | | | | | | |
| Acupuncture:BT | 0 | No concerns | Low risk | No concern | Major concerns | No concerns | Major concerns | Low | Imprecision, Incoherence |
| Acupuncture:EX | 0 | No concerns | Low risk | No concern | Major concerns | No concerns | Major concerns | Low | Imprecision, Incoherence |
| Acupuncture:Herb | 0 | No concerns | Low risk | No concern | Major concerns | No concerns | Major concerns | Low | Imprecision, Incoherence |
| Acupuncture:MBSR | 0 | No concerns | Low risk | No concern | Major concerns | No concerns | Major concerns | Low | Imprecision, Incoherence |
| Acupuncture:SH | 0 | Major concerns | Low risk | No concern | Major concerns | No concerns | Major concerns | Low | Within-study bias, Imprecision, Incoherence |
| Acupuncture:TAU | 0 | Major concerns | Low risk | No concern | Major concerns | No concerns | Major concerns | Low | Within-study bias, Imprecision, Incoherence |
| Acupuncture:dCBTi | 0 | Major concerns | Low risk | No concern | Some concerns | Some concerns | Major concerns | Very low | Within-study bias, Imprecision, Heterogeneity, Incoherence |
| Acupuncture:gCBTi | 0 | Some concerns | Low risk | No concern | Some concerns | Some concerns | Major concerns | Very low | Within-study bias, Imprecision, Heterogeneity, Incoherence |
| Acupuncture:rtms | 0 | No concerns | Low risk | No concern | Major concerns | No concerns | Major concerns | Low | Imprecision, Incoherence |
| Acupuncture:sCBTi | 0 | Major concerns | Low risk | No concern | Major concerns | No concerns | Major concerns | Very low | Within-study bias, Imprecision, Incoherence |
| Acupuncture:stepcare | 0 | No concerns | Low risk | No concern | Major concerns | No concerns | Major concerns | Low | Imprecision, Incoherence |
| Acupuncture:tCBTi | 0 | Major concerns | Low risk | No concern | Major concerns | No concerns | Major concerns | Very low | Within-study bias, Imprecision, Incoherence |
| BT:EX | 0 | Some concerns | Low risk | No concern | Major concerns | No concerns | Major concerns | Very low | Within-study bias, Imprecision, Incoherence |
| BT:Herb | 0 | Some concerns | Low risk | No concern | Major concerns | No concerns | Major concerns | Very low | Within-study bias, Imprecision, Incoherence |
| BT:MBSR | 0 | Some concerns | Low risk | No concern | Major concerns | No concerns | Major concerns | Very low | Within-study bias, Imprecision, Incoherence |
| BT:SH | 0 | Some concerns | Low risk | No concern | Major concerns | No concerns | Major concerns | Very low | Within-study bias, Imprecision, Incoherence |
| BT:TAU | 0 | Some concerns | Low risk | No concern | Major concerns | No concerns | Major concerns | Very low | Within-study bias, Imprecision, Incoherence |
| BT:dCBTi | 0 | Some concerns | Low risk | No concern | Major concerns | No concerns | Major concerns | Very low | Within-study bias, Imprecision, Incoherence |
| BT:gCBTi | 0 | Some concerns | Low risk | No concern | Some concerns | Some concerns | Major concerns | Very low | Within-study bias, Imprecision, Heterogeneity, Incoherence |
| BT:rtms | 0 | Some concerns | Low risk | No concern | Major concerns | No concerns | Major concerns | Very low | Within-study bias, Imprecision, Incoherence |
| BT:sCBTi | 0 | Some concerns | Low risk | No concern | Major concerns | No concerns | Major concerns | Very low | Within-study bias, Imprecision, Incoherence |
| BT:stepcare | 0 | No concerns | Low risk | No concern | Major concerns | No concerns | Major concerns | Low | Imprecision, Incoherence |
| BT:tCBTi | 0 | Some concerns | Low risk | No concern | Major concerns | No concerns | Major concerns | Very low | Within-study bias, Imprecision, Incoherence |
| EX:Herb | 0 | Some concerns | Low risk | No concern | Major concerns | No concerns | Major concerns | Very low | Within-study bias, Imprecision, Incoherence |
| EX:MBSR | 0 | No concerns | Low risk | No concern | Major concerns | No concerns | Major concerns | Low | Imprecision, Incoherence |
| EX:SH | 0 | Major concerns | Low risk | No concern | Major concerns | No concerns | Major concerns | Very low | Within-study bias, Imprecision, Incoherence |
| EX:TAU | 0 | No concerns | Low risk | No concern | Major concerns | No concerns | Major concerns | Low | Imprecision, Incoherence |
| EX:dCBTi | 0 | No concerns | Low risk | No concern | Major concerns | No concerns | Major concerns | Low | Imprecision, Incoherence |
| EX:gCBTi | 0 | Some concerns | Low risk | No concern | Some concerns | Some concerns | Major concerns | Low | Within-study bias, Imprecision, Heterogeneity, Incoherence |
| EX:iCBTi | 0 | Major concerns | Low risk | No concern | Major concerns | No concerns | Major concerns | Very low | Within-study bias, Imprecision, Incoherence |
| EX:rtms | 0 | Major concerns | Low risk | No concern | Major concerns | No concerns | Major concerns | Very low | Within-study bias, Imprecision, Incoherence |
| EX:stepcare | 0 | No concerns | Low risk | No concern | Major concerns | No concerns | Major concerns | Low | Imprecision, Incoherence |
| EX:tCBTi | 0 | Some concerns | Low risk | No concern | Major concerns | No concerns | Major concerns | Very low | Within-study bias, Imprecision, Incoherence |
| Herb:MBSR | 0 | Some concerns | Low risk | No concern | Major concerns | No concerns | Major concerns | Very low | Within-study bias, Imprecision, Incoherence |
| Herb:SH | 0 | Some concerns | Low risk | No concern | Major concerns | No concerns | Major concerns | Very low | Within-study bias, Imprecision, Incoherence |
| Herb:TAU | 0 | Some concerns | Low risk | No concern | Some concerns | Some concerns | Major concerns | Low | Within-study bias, Imprecision, Heterogeneity, Incoherence |
| Herb:dCBTi | 0 | Some concerns | Low risk | No concern | Major concerns | No concerns | Major concerns | Very low | Within-study bias, Imprecision, Incoherence |
| Herb:gCBTi | 0 | Some concerns | Low risk | No concern | Major concerns | No concerns | Major concerns | Very low | Within-study bias, Imprecision, Incoherence |
| Herb:rtms | 0 | Some concerns | Low risk | No concern | Major concerns | No concerns | Major concerns | Very low | Within-study bias, Imprecision, Incoherence |
| Herb:sCBTi | 0 | Some concerns | Low risk | No concern | Major concerns | No concerns | Major concerns | Low | Within-study bias, Imprecision, Incoherence |
| Herb:stepcare | 0 | No concerns | Low risk | No concern | Major concerns | No concerns | Major concerns | Low | Imprecision, Incoherence |
| Herb:tCBTi | 0 | Some concerns | Low risk | No concern | Major concerns | No concerns | Major concerns | Low | Within-study bias, Imprecision, Incoherence |
| MBSR:SH | 0 | Some concerns | Low risk | No concern | Major concerns | No concerns | Major concerns | Low | Within-study bias, Imprecision, Incoherence |
| MBSR:TAU | 0 | No concerns | Low risk | No concern | Some concerns | Some concerns | Major concerns | Moderate | Imprecision, Heterogeneity, Incoherence |
| MBSR:dCBTi | 0 | Some concerns | Low risk | No concern | Major concerns | No concerns | Major concerns | Low | Within-study bias, Imprecision, Incoherence |
| MBSR:iCBTi | 0 | Some concerns | Low risk | No concern | Major concerns | No concerns | Major concerns | Low | Within-study bias, Imprecision, Incoherence |
| MBSR:rtms | 0 | Major concerns | Low risk | No concern | Major concerns | No concerns | Major concerns | Very low | Within-study bias, Imprecision, Incoherence |
| MBSR:sCBTi | 0 | Some concerns | Low risk | No concern | Major concerns | No concerns | Major concerns | Low | Within-study bias, Imprecision, Incoherence |
| MBSR:stepcare | 0 | No concerns | Low risk | No concern | Major concerns | No concerns | Major concerns | Low | Imprecision, Incoherence |
| MBSR:tCBTi | 0 | Some concerns | Low risk | No concern | Major concerns | No concerns | Major concerns | Low | Within-study bias, Imprecision, Incoherence |
| SH:TAU | 0 | Major concerns | Low risk | No concern | Major concerns | No concerns | Major concerns | Very low | Within-study bias, Imprecision, Incoherence |
| SH:dCBTi | 0 | Major concerns | Low risk | No concern | Some concerns | Some concerns | Major concerns | Very low | Within-study bias, Imprecision, Heterogeneity, Incoherence |
| SH:gCBTi | 0 | Some concerns | Low risk | No concern | No concerns | Major concerns | Major concerns | High | Within-study bias, Heterogeneity, Incoherence |
| SH:rtms | 0 | Major concerns | Low risk | No concern | Major concerns | No concerns | Major concerns | Very low | Within-study bias, Imprecision, Incoherence |
| SH:sCBTi | 0 | Major concerns | Low risk | No concern | Major concerns | No concerns | Major concerns | Very low | Within-study bias, Imprecision, Incoherence |
| SH:stepcare | 0 | Major concerns | Low risk | No concern | Major concerns | No concerns | Major concerns | Very low | Within-study bias, Imprecision, Incoherence |
| TAU:rtms | 0 | Major concerns | Low risk | No concern | Major concerns | No concerns | Major concerns | Very low | Within-study bias, Imprecision, Incoherence |
| TAU:stepcare | 0 | Major concerns | Low risk | No concern | Major concerns | No concerns | Major concerns | Very low | Within-study bias, Imprecision, Incoherence |
| dCBTi:gCBTi | 0 | Some concerns | Low risk | No concern | Major concerns | No concerns | Major concerns | Low | Within-study bias, Imprecision, Incoherence |
| dCBTi:iCBTi | 0 | Major concerns | Low risk | No concern | Some concerns | Some concerns | Major concerns | Very low | Within-study bias, Imprecision, Heterogeneity, Incoherence |
| dCBTi:rtms | 0 | Major concerns | Low risk | No concern | Major concerns | No concerns | Major concerns | Very low | Within-study bias, Imprecision, Incoherence |
| dCBTi:stepcare | 0 | Major concerns | Low risk | No concern | Major concerns | No concerns | Major concerns | Very low | Within-study bias, Imprecision, Incoherence |
| dCBTi:tCBTi | 0 | Some concerns | Low risk | No concern | Major concerns | No concerns | Major concerns | Low | Within-study bias, Imprecision, Incoherence |
| gCBTi:iCBTi | 0 | Some concerns | Low risk | No concern | Some concerns | Some concerns | Major concerns | Low | Within-study bias, Imprecision, Heterogeneity, Incoherence |
| gCBTi:rtms | 0 | Major concerns | Low risk | No concern | Major concerns | No concerns | Major concerns | Very low | Within-study bias, Imprecision, Incoherence |
| gCBTi:sCBTi | 0 | Some concerns | Low risk | No concern | No concerns | Major concerns | Major concerns | Low | Within-study bias, Heterogeneity, Incoherence |
| gCBTi:stepcare | 0 | Some concerns | Low risk | No concern | Some concerns | Some concerns | Major concerns | Low | Within-study bias, Imprecision, Heterogeneity, Incoherence |
| gCBTi:tCBTi | 0 | Some concerns | Low risk | No concern | Major concerns | No concerns | Major concerns | Low | Within-study bias, Imprecision, Incoherence |
| iCBTi:tCBTi | 0 | Major concerns | Low risk | No concern | Some concerns | Some concerns | Major concerns | Very low | Within-study bias, Imprecision, Heterogeneity, Incoherence |
| rtms:sCBTi | 0 | Major concerns | Low risk | No concern | Major concerns | No concerns | Major concerns | Very low | Within-study bias, Imprecision, Incoherence |
| rtms:stepcare | 0 | No concerns | Low risk | No concern | Major concerns | No concerns | Major concerns | Low | Imprecision, Incoherence |
| rtms:tCBTi | 0 | Major concerns | Low risk | No concern | Major concerns | No concerns | Major concerns | Very low | Within-study bias, Imprecision, Incoherence |
| sCBTi:stepcare | 0 | Major concerns | Low risk | No concern | Major concerns | No concerns | Major concerns | Very low | Within-study bias, Imprecision |
| sCBTi:tCBTi | 0 | Some concerns | Low risk | No concern | Some concerns | Some concerns | Major concerns | Low | Within-study bias, Imprecision, Heterogeneity, Incoherence |
| stepcare:tCBTi | 0 | Major concerns | Low risk | No concern | Major concerns | No concerns | Major concerns | Very low | Within-study bias, Imprecision, Incoherence |

Abbreviations: BT = behavioral therapy, dCBTi = digital CBTi, EX = exercise, gCBTi = group CBTi, iCBTi = individual CBTi, ISI = insomnia sleep index, MBSR = mindfulness-based stress reduction, rTMS = repetitive transcranial magnetic stimulation, sCBTi = self-help CBTi, SE = sleep efficiency, SH = sleep hygiene, SOL = sleep onset latency, stepcare = stepped-care CBTi, TAU = treatment as usual, tCBTi = teleCBTi, TST = total sleep time, and WASO = wake after sleep onset

**Supplementary Material 16** Certainty of evidence evaluating by CINeMA for SOL

| Comparison | Number of studies | Within-study bias | Reporting bias | Indirectness | Imprecision | Heterogeneity | Incoherence | Confidence rating | Reason(s) for downgrading |
| --- | --- | --- | --- | --- | --- | --- | --- | --- | --- |
| **Mixed evidence** | | | | | | | | | |
| Acupuncture:iCBTi | 1 | No concerns | Low risk | No concern | No concerns | No concerns | No concerns | High |  |
| BT:iCBTi | 1 | Some concerns | Low risk | No concern | Some concerns | No concerns | No concerns | Moderate | Within-study bias, Imprecision |
| EX:gCBTi | 1 | No concerns | Low risk | No concern | No concerns | Some concerns | No concerns | Moderate | Heterogeneity |
| EX:sCBTi | 1 | No concerns | Low risk | No concern | No concerns | Major concerns | No concerns | Moderate | Heterogeneity |
| MBSR:gCBTi | 1 | No concerns | Low risk | No concern | No concerns | No concerns | No concerns | High |  |
| TAU:dCBTi | 1 | Some concerns | Low risk | No concern | No concerns | No concerns | No concerns | Moderate | Within-study bias |
| TAU:gCBTi | 3 | Some concerns | Low risk | No concern | No concerns | No concerns | No concerns | Moderate | Within-study bias |
| TAU:iCBTi | 1 | Major concerns | Low risk | No concern | No concerns | Some concerns | No concerns | Low | Within-study bias, Heterogeneity |
| TAU:sCBTi | 4 | No concerns | Low risk | No concern | No concerns | No concerns | No concerns | High |  |
| dCBTi:sCBTi | 1 | Some concerns | Low risk | No concern | No concerns | No concerns | No concerns | Moderate | Within-study bias |
| iCBTi:sCBTi | 1 | Major concerns | Low risk | No concern | No concerns | Some concerns | No concerns | Low | Within-study bias, Heterogeneity |
| iCBTi:stepcare | 1 | No concerns | Low risk | No concern | No concerns | Some concerns | No concerns | Moderate | Heterogeneity |
| **Indirect evidence** | | | | | | | | | |
| Acupuncture:BT | 0 | No concerns | Low risk | No concern | Major concerns | No concerns | No concerns | Moderate | Imprecision |
| Acupuncture:EX | 0 | No concerns | Low risk | No concern | No concerns | Some concerns | No concerns | Moderate | Heterogeneity |
| Acupuncture:MBSR | 0 | No concerns | Low risk | No concern | Some concerns | Some concerns | No concerns | Moderate | Imprecision, Heterogeneity |
| Acupuncture:TAU | 0 | No concerns | Low risk | No concern | Some concerns | No concerns | No concerns | Moderate | Imprecision |
| Acupuncture:dCBTi | 0 | No concerns | Low risk | No concern | No concerns | No concerns | No concerns | High |  |
| Acupuncture:gCBTi | 0 | No concerns | Low risk | No concern | No concerns | No concerns | No concerns | High |  |
| Acupuncture:sCBTi | 0 | No concerns | Low risk | No concern | No concerns | Some concerns | No concerns | High | Heterogeneity |
| Acupuncture:stepcare | 0 | No concerns | Low risk | No concern | Some concerns | No concerns | No concerns | Moderate | Imprecision |
| BT:EX | 0 | Some concerns | Low risk | No concern | Some concerns | No concerns | No concerns | Moderate | Within-study bias, Imprecision |
| BT:MBSR | 0 | No concerns | Low risk | No concern | Major concerns | No concerns | No concerns | Moderate | Imprecision |
| BT:TAU | 0 | Some concerns | Low risk | No concern | Major concerns | No concerns | No concerns | Low | Within-study bias, Imprecision |
| BT:dCBTi | 0 | Some concerns | Low risk | No concern | No concerns | No concerns | No concerns | Moderate | Within-study bias |
| BT:gCBTi | 0 | Some concerns | Low risk | No concern | No concerns | No concerns | No concerns | Moderate | Within-study bias |
| BT:sCBTi | 0 | Some concerns | Low risk | No concern | Some concerns | No concerns | No concerns | Moderate | Within-study bias, Imprecision |
| BT:stepcare | 0 | No concerns | Low risk | No concern | Some concerns | No concerns | No concerns | Moderate | Imprecision |
| EX:MBSR | 0 | No concerns | Low risk | No concern | Some concerns | No concerns | No concerns | Moderate | Imprecision |
| EX:TAU | 0 | No concerns | Low risk | No concern | No concerns | Some concerns | No concerns | Moderate | Heterogeneity |
| EX:dCBTi | 0 | Some concerns | Low risk | No concern | No concerns | Some concerns | No concerns | Moderate | Within-study bias, Heterogeneity |
| EX:iCBTi | 0 | No concerns | Low risk | No concern | Some concerns | Some concerns | No concerns | Moderate | Imprecision, Heterogeneity |
| EX:stepcare | 0 | No concerns | Low risk | No concern | Some concerns | Some concerns | No concerns | Moderate | Imprecision, Heterogeneity |
| MBSR:TAU | 0 | No concerns | Low risk | No concern | Some concerns | Some concerns | No concerns | Moderate | Imprecision, Heterogeneity |
| MBSR:dCBTi | 0 | No concerns | Low risk | No concern | No concerns | No concerns | No concerns | High |  |
| MBSR:iCBTi | 0 | No concerns | Low risk | No concern | Some concerns | No concerns | No concerns | Moderate | Imprecision |
| MBSR:sCBTi | 0 | No concerns | Low risk | No concern | Some concerns | No concerns | No concerns | Moderate | Imprecision |
| MBSR:stepcare | 0 | No concerns | Low risk | No concern | Major concerns | No concerns | No concerns | Moderate | Imprecision |
| TAU:stepcare | 0 | No concerns | Low risk | No concern | Some concerns | No concerns | No concerns | Moderate | Imprecision |
| dCBTi:gCBTi | 0 | Some concerns | Low risk | No concern | Some concerns | No concerns | No concerns | Moderate | Within-study bias, Imprecision |
| dCBTi:iCBTi | 0 | Major concerns | Low risk | No concern | No concerns | No concerns | No concerns | Moderate | Within-study bias |
| dCBTi:stepcare | 0 | No concerns | Low risk | No concern | No concerns | Some concerns | No concerns | Moderate | Heterogeneity |
| gCBTi:iCBTi | 0 | Major concerns | Low risk | No concern | No concerns | Some concerns | No concerns | Moderate | Imprecision, Heterogeneity |
| gCBTi:sCBTi | 0 | No concerns | Low risk | No concern | No concerns | No concerns | No concerns | High |  |
| gCBTi:stepcare | 0 | No concerns | Low risk | No concern | No concerns | Some concerns | No concerns | Moderate | Heterogeneity |
| sCBTi:stepcare | 0 | No concerns | Low risk | No concern | Some concerns | Some concerns | No concerns | Moderate | Imprecision, Heterogeneity |

Abbreviations: BT = behavioral therapy, dCBTi = digital CBTi, EX = exercise, gCBTi = group CBTi, iCBTi = individual CBTi, ISI = insomnia sleep index, MBSR = mindfulness-based stress reduction, rTMS = repetitive transcranial magnetic stimulation, sCBTi = self-help CBTi, SE = sleep efficiency, SH = sleep hygiene, SOL = sleep onset latency, stepcare = stepped-care CBTi, TAU = treatment as usual, tCBTi = teleCBTi, TST = total sleep time, and WASO = wake after sleep onset

**Supplementary Material 17** Certainty of evidence evaluating by CINeMA for WASO

| Comparison | Number of studies | Within-study bias | Reporting bias | Indirectness | Imprecision | Heterogeneity | Incoherence | Confidence rating | Reason(s) for downgrading |
| --- | --- | --- | --- | --- | --- | --- | --- | --- | --- |
| **Mixed evidence** | | | | | | | | | |
| Acupunture:iCBTi | 1 | No concerns | Low risk | No concern | Some concerns | Some concerns | Some concerns | Moderate | Imprecision Heterogeneity, Incoherence |
| BT:iCBTi | 1 | Some concerns | Low risk | No concern | Some concerns | Some concerns | Some concerns | Low | Within-study bias, Imprecision, Heterogeneity, Incoherence |
| EX:gCBTi | 1 | No concerns | Low risk | No concern | Some concerns | No concerns | No concerns | Moderate | Imprecision |
| EX:sCBTi | 1 | No concerns | Low risk | No concern | Some concerns | Some concerns | No concerns | Moderate | Imprecision, Heterogeneity |
| MBSR:gCBTi | 1 | No concerns | Low risk | No concern | Some concerns | No concerns | Some concerns | Moderate | Imprecision, Incoherence |
| TAU:dCBTi | 1 | Some concerns | Low risk | No concern | No concerns | No concerns | No concerns | Moderate | Within-study bias |
| TAU:gCBTi | 3 | Some concerns | Low risk | No concern | No concerns | No concerns | No concerns | Moderate | Within-study bias |
| TAU:iCBTi | 1 | Major concerns | Low risk | No concern | No concerns | Some concerns | No concerns | Low | Within-study bias, Heterogeneity |
| TAU:sCBTi | 4 | No concerns | Low risk | No concern | No concerns | Some concerns | No concerns | Moderate | Heterogeneity |
| dCBTi:sCBTi | 1 | Some concerns | Low risk | No concern | Some concerns | No concerns | No concerns | Moderate | Within-study bias, Imprecision |
| iCBTi:sCBTi | 1 | Major concerns | Low risk | No concern | Some concerns | Some concerns | No concerns | Low | Within-study bias, Imprecision, Heterogeneity |
| iCBTi:stepcare | 1 | No concerns | Low risk | No concern | Some concerns | No concerns | Some concerns | Moderate | Imprecision, Incoherence |
| **Indirect evidence** | | | | | | | | | |
| Acupunture:BT | 0 | No concerns | Low risk | No concern | Major concerns | No concerns | Some concerns | Low | Imprecision, Incoherence |
| Acupunture:EX | 0 | No concerns | Low risk | No concern | Some concerns | Some concerns | Some concerns | Low | Imprecision, Heterogeneity, Incoherence |
| Acupunture:MBSR | 0 | No concerns | Low risk | No concern | Major concerns | No concerns | Some concerns | Low | Imprecision, Incoherence |
| Acupunture:TAU | 0 | No concerns | Low risk | No concern | Some concerns | Some concerns | Some concerns | Low | Imprecision, Heterogeneity, Incoherence |
| Acupunture:dCBTi | 0 | No concerns | Low risk | No concern | Some concerns | Some concerns | Some concerns | Low | Imprecision, Heterogeneity, Incoherence |
| Acupunture:gCBTi | 0 | No concerns | Low risk | No concern | No concerns | Some concerns | Some concerns | Moderate | Heterogeneity, Incoherence |
| Acupunture:sCBTi | 0 | No concerns | Low risk | No concern | Some concerns | Some concerns | Some concerns | Moderate | Imprecision, Heterogeneity, Incoherence |
| Acupunture:stepcare | 0 | No concerns | Low risk | No concern | Major concerns | No concerns | Some concerns | Low | Imprecision, Incoherence |
| BT:EX | 0 | Some concerns | Low risk | No concern | Major concerns | No concerns | Some concerns | Low | Within-study bias, Imprecision, Incoherence |
| BT:MBSR | 0 | No concerns | Low risk | No concern | Major concerns | No concerns | Some concerns | Low | Imprecision, Incoherence |
| BT:TAU | 0 | Some concerns | Low risk | No concern | Major concerns | No concerns | Some concerns | Low | Within-study bias, Imprecision, Incoherence |
| BT:dCBTi | 0 | Some concerns | Low risk | No concern | Some concerns | Some concerns | Some concerns | Low | Within-study bias, Imprecision, Heterogeneity, Incoherence |
| BT:gCBTi | 0 | Some concerns | Low risk | No concern | Some concerns | No concerns | Some concerns | Moderate | Within-study bias, Imprecision, Incoherence |
| BT:sCBTi | 0 | Some concerns | Low risk | No concern | Major concerns | No concerns | Some concerns | Low | Within-study bias, Imprecision, Incoherence |
| BT:stepcare | 0 | No concerns | Low risk | No concern | Major concerns | No concerns | Some concerns | Low | Imprecision, Incoherence |
| EX:MBSR | 0 | No concerns | Low risk | No concern | Major concerns | No concerns | Some concerns | Low | Imprecision, Incoherence |
| EX:TAU | 0 | No concerns | Low risk | No concern | No concerns | Some concerns | Some concerns | Moderate | Heterogeneity, Incoherence |
| EX:dCBTi | 0 | Some concerns | Low risk | No concern | Some concerns | Some concerns | Some concerns | Low | Within-study bias, Imprecision, Heterogeneity, Incoherence |
| EX:iCBTi | 0 | No concerns | Low risk | No concern | Major concerns | No concerns | Some concerns | Moderate | Imprecision, Incoherence |
| EX:stepcare | 0 | No concerns | Low risk | No concern | Some concerns | Some concerns | Some concerns | Moderate | Imprecision, Heterogeneity, Incoherence |
| MBSR:TAU | 0 | No concerns | Low risk | No concern | Some concerns | No concerns | Some concerns | Moderate | Imprecision, Incoherence |
| MBSR:dCBTi | 0 | No concerns | Low risk | No concern | Major concerns | No concerns | Some concerns | Low | Imprecision, Incoherence |
| MBSR:iCBTi | 0 | No concerns | Low risk | No concern | Major concerns | No concerns | Some concerns | Low | Imprecision, Incoherence |
| MBSR:sCBTi | 0 | No concerns | Low risk | No concern | Major concerns | No concerns | Some concerns | Low | Imprecision, Incoherence |
| MBSR:stepcare | 0 | No concerns | Low risk | No concern | Some concerns | Some concerns | Some concerns | Moderate | Imprecision, Heterogeneity, Incoherence |
| TAU:stepcare | 0 | No concerns | Low risk | No concern | Major concerns | No concerns | Some concerns | Low | Imprecision, Incoherence |
| dCBTi:gCBTi | 0 | Some concerns | Low risk | No concern | Some concerns | Some concerns | Some concerns | Low | Within-study bias, Imprecision, Heterogeneity, Incoherence |
| dCBTi:iCBTi | 0 | Some concerns | Low risk | No concern | Some concerns | Some concerns | Some concerns | Low | Within-study bias, Imprecision, Heterogeneity, Incoherence |
| dCBTi:stepcare | 0 | No concerns | Low risk | No concern | Some concerns | No concerns | Some concerns | Moderate | Imprecision, Incoherence |
| gCBTi:iCBTi | 0 | Major concerns | Low risk | No concern | Some concerns | No concerns | Some concerns | Low | Within-study bias, Imprecision, Incoherence |
| gCBTi:sCBTi | 0 | No concerns | Low risk | No concern | No concerns | Some concerns | Some concerns | Moderate | Heterogeneity, Incoherence |
| gCBTi:stepcare | 0 | No concerns | Low risk | No concern | No concerns | Some concerns | Some concerns | Moderate | Heterogeneity, Incoherence |
| sCBTi:stepcare | 0 | No concerns | Low risk | No concern | Some concerns | Some concerns | Some concerns | Moderate | Imprecision, Heterogeneity, Incoherence |

Abbreviations: BT = behavioral therapy, dCBTi = digital CBTi, EX = exercise, gCBTi = group CBTi, iCBTi = individual CBTi, ISI = insomnia sleep index, MBSR = mindfulness-based stress reduction, rTMS = repetitive transcranial magnetic stimulation, sCBTi = self-help CBTi, SE = sleep efficiency, SH = sleep hygiene, SOL = sleep onset latency, stepcare = stepped-care CBTi, TAU = treatment as usual, tCBTi = teleCBTi, TST = total sleep time, and WASO = wake after sleep onset

**Supplementary Material 18** Certainty of evidence evaluating by CINeMA for TST

| Comparison | Number of studies | Within-study bias | Reporting bias | Indirectness | Imprecision | Heterogeneity | Incoherence | Confidence rating | Reason(s) for downgrading |
| --- | --- | --- | --- | --- | --- | --- | --- | --- | --- |
| **Mixed evidence** | | | | | | | | | |
| Acupunture:iCBTi | 1 | No concerns | Low risk | No concern | Some concerns | No concerns | No concerns | Moderate | Imprecision |
| BT:iCBTi | 1 | Some concerns | Low risk | No concern | No concerns | Some concerns | No concerns | Moderate | Within-study bias, Heterogeneity |
| EX:gCBTi | 1 | No concerns | Low risk | No concern | No concerns | Major concerns | No concerns | Moderate | Heterogeneity |
| EX:sCBTi | 1 | No concerns | Low risk | No concern | No concerns | Major concerns | No concerns | Moderate | Heterogeneity |
| MBSR:gCBTi | 1 | No concerns | Low risk | No concern | Some concerns | No concerns | No concerns | Moderate | Imprecision |
| TAU:dCBTi | 1 | Some concerns | Low risk | No concern | No concerns | Some concerns | Some concerns | Low | Within-study bias, Heterogeneity, Incoherence |
| TAU:gCBTi | 3 | Some concerns | Low risk | No concern | No concerns | Some concerns | No concerns | Moderate | Within-study bias, Heterogeneity |
| TAU:iCBTi | 1 | Major concerns | Low risk | No concern | No concerns | Some concerns | No concerns | Low | Within-study bias, Heterogeneity |
| TAU:sCBTi | 4 | No concerns | Low risk | No concern | No concerns | Some concerns | No concerns | Moderate | Heterogeneity |
| dCBTi:sCBTi | 1 | Some concerns | Low risk | No concern | Some concerns | No concerns | Major concerns | Low | Within-study bias, Imprecision, Incoherence |
| iCBTi:sCBTi | 1 | Major concerns | Low risk | No concern | Some concerns | No concerns | No concerns | Low | Within-study bias, Imprecision |
| iCBTi:stepcare | 1 | No concerns | Low risk | No concern | Some concerns | No concerns | No concerns | Moderate | Imprecision |
| **Indirect evidence** | | | | | | | | | |
| Acupunture:BT | 0 | No concerns | Low risk | No concern | Some concerns | Some concerns | No concerns | Moderate | Imprecision, Heterogeneity |
| Acupunture:EX | 0 | No concerns | Low risk | No concern | Major concerns | No concerns | No concerns | Moderate | Imprecision |
| Acupunture:MBSR | 0 | No concerns | Low risk | No concern | Major concerns | No concerns | No concerns | Moderate | Imprecision |
| Acupunture:TAU | 0 | No concerns | Low risk | No concern | Some concerns | No concerns | No concerns | Moderate | Imprecision |
| Acupunture:dCBTi | 0 | No concerns | Low risk | No concern | Some concerns | Some concerns | No concerns | Moderate | Imprecision, Heterogeneity |
| Acupunture:gCBTi | 0 | No concerns | Low risk | No concern | Major concerns | No concerns | No concerns | Moderate | Imprecision |
| Acupunture:sCBTi | 0 | No concerns | Low risk | No concern | Some concerns | Some concerns | No concerns | Moderate | Imprecision, Heterogeneity |
| Acupunture:stepcare | 0 | No concerns | Low risk | No concern | Major concerns | No concerns | No concerns | Moderate | Imprecision |
| BT:EX | 0 | Some concerns | Low risk | No concern | Some concerns | Some concerns | No concerns | Low | Within-study bias, Imprecision, Heterogeneity |
| BT:MBSR | 0 | No concerns | Low risk | No concern | Some concerns | Some concerns | No concerns | Moderate | Imprecision, Heterogeneity |
| BT:TAU | 0 | Some concerns | Low risk | No concern | Some concerns | No concerns | No concerns | Moderate | Within-study bias, Imprecision |
| BT:dCBTi | 0 | Some concerns | Low risk | No concern | Major concerns | No concerns | No concerns | Low | Within-study bias, Imprecision |
| BT:gCBTi | 0 | Some concerns | Low risk | No concern | Some concerns | Some concerns | No concerns | Low | Within-study bias, Imprecision, Heterogeneity |
| BT:sCBTi | 0 | Some concerns | Low risk | No concern | Some concerns | Some concerns | No concerns | Low | Within-study bias, Imprecision, Heterogeneity |
| BT:stepcare | 0 | No concerns | Low risk | No concern | Some concerns | Some concerns | No concerns | Moderate | Imprecision, Heterogeneity |
| EX:MBSR | 0 | No concerns | Low risk | No concern | Some concerns | Some concerns | No concerns | Moderate | Imprecision, Heterogeneity |
| EX:TAU | 0 | No concerns | Low risk | No concern | Some concerns | No concerns | No concerns | Moderate | ["Imprecision"] |
| EX:dCBTi | 0 | Some concerns | Low risk | No concern | Some concerns | Some concerns | No concerns | Low | Within-study bias, Imprecision, Heterogeneity |
| EX:iCBTi | 0 | No concerns | Low risk | No concern | Some concerns | No concerns | No concerns | Moderate | Imprecision |
| EX:stepcare | 0 | No concerns | Low risk | No concern | Major concerns | No concerns | No concerns | Moderate | Imprecision |
| MBSR:TAU | 0 | No concerns | Low risk | No concern | Some concerns | Some concerns | No concerns | Moderate | Imprecision, Heterogeneity |
| MBSR:dCBTi | 0 | No concerns | Low risk | No concern | Some concerns | No concerns | No concerns | Moderate | Imprecision |
| MBSR:iCBTi | 0 | No concerns | Low risk | No concern | Some concerns | Some concerns | No concerns | Moderate | Imprecision, Heterogeneity |
| MBSR:sCBTi | 0 | No concerns | Low risk | No concern | Some concerns | Some concerns | No concerns | Moderate | Imprecision, Heterogeneity |
| MBSR:stepcare | 0 | No concerns | Low risk | No concern | Major concerns | No concerns | No concerns | Moderate | Imprecision |
| TAU:stepcare | 0 | No concerns | Low risk | No concern | Some concerns | No concerns | No concerns | Moderate | Imprecision |
| dCBTi:gCBTi | 0 | Some concerns | Low risk | No concern | Some concerns | No concerns | No concerns | Moderate | Within-study bias, Imprecision |
| dCBTi:iCBTi | 0 | Major concerns | Low risk | No concern | No concerns | Some concerns | No concerns | Low | Within-study bias, Heterogeneity |
| dCBTi:stepcare | 0 | No concerns | Low risk | No concern | Some concerns | Some concerns | No concerns | Moderate | Imprecision, Heterogeneity |
| gCBTi:iCBTi | 0 | Major concerns | Low risk | No concern | Some concerns | No concerns | No concerns | Low | Within-study bias, Imprecision |
| gCBTi:sCBTi | 0 | No concerns | Low risk | No concern | No concerns | Some concerns | No concerns | Moderate | Heterogeneity |
| gCBTi:stepcare | 0 | No concerns | Low risk | No concern | Major concerns | No concerns | No concerns | Moderate | Imprecision |
| sCBTi:stepcare | 0 | No concerns | Low risk | No concern | Major concerns | No concerns | No concerns | Moderate | Imprecision |

Abbreviations: BT = behavioral therapy, dCBTi = digital CBTi, EX = exercise, gCBTi = group CBTi, iCBTi = individual CBTi, ISI = insomnia sleep index, MBSR = mindfulness-based stress reduction, rTMS = repetitive transcranial magnetic stimulation, sCBTi = self-help CBTi, SE = sleep efficiency, SH = sleep hygiene, SOL = sleep onset latency, stepcare = stepped-care CBTi, TAU = treatment as usual, tCBTi = teleCBTi, TST = total sleep time, and WASO = wake after sleep onset

**Supplementary Material 15** Certainty of evidence evaluating by CINeMA for SE

| Comparison | Number of studies | Within-study bias | Reporting bias | Indirectness | Imprecision | Heterogeneity | Incoherence | Confidence rating | Reason(s) for downgrading |
| --- | --- | --- | --- | --- | --- | --- | --- | --- | --- |
| **Mixed evidence** | | | | | | | | | |
| Acupunture:iCBTi | 1 | No concerns | Low risk | No concern | Major concerns | No concerns | Major concerns | Low | Imprecision, Incoherence |
| BT:iCBTi | 1 | Some concerns | Low risk | No concern | Major concerns | No concerns | Major concerns | Very low | Within-study bias, Imprecision, Incoherence |
| EX:gCBTi | 1 | No concerns | Low risk | No concern | Major concerns | No concerns | No concerns | Moderate | Imprecision |
| EX:sCBTi | 1 | No concerns | Low risk | No concern | Major concerns | No concerns | No concerns | Moderate | Imprecision |
| MBSR:gCBTi | 1 | No concerns | Low risk | No concern | Major concerns | No concerns | Major concerns | Low | Imprecision, Incoherence |
| SH:tCBTi | 1 | Major concerns | Low risk | No concern | Major concerns | No concerns | Major concerns | Very low | Within-study bias, Imprecision, Incoherence |
| TAU:dCBTi | 1 | Some concerns | Low risk | No concern | No concerns | No concerns | Major concerns | Low | Within-study bias, Incoherence |
| TAU:gCBTi | 3 | Some concerns | Low risk | No concern | Some concerns | Some concerns | No concerns | Low | Within-study bias, Imprecision, Heterogeneity |
| TAU:iCBTi | 1 | Major concerns | Low risk | No concern | Major concerns | No concerns | No concerns | Low | Within-study bias, Imprecision |
| TAU:sCBTi | 4 | Some concerns | Low risk | No concern | Major concerns | No concerns | Major concerns | Very low | Within-study bias, Imprecision, Incoherence |
| TAU:tCBTi | 1 | Some concerns | Low risk | No concern | Major concerns | No concerns | Major concerns | Very low | Within-study bias, Imprecision, Incoherence |
| dCBTi:sCBTi | 1 | Some concerns | Low risk | No concern | No concerns | No concerns | Major concerns | Low | Within-study bias, Incoherence |
| iCBTi:sCBTi | 1 | Major concerns | Low risk | No concern | Major concerns | No concerns | No concerns | Low | Within-study bias, Imprecision |
| iCBTi:stepcare | 1 | No concerns | Low risk | No concern | Major concerns | No concerns | Major concerns | Low | Imprecision, Incoherence |
| **Indirect evidence** | | | | | | | | | |
| Acupunture:BT | 0 | No concerns | Low risk | No concern | Major concerns | No concerns | Major concerns | Low | Imprecision, Incoherence |
| Acupunture:EX | 0 | No concerns | Low risk | No concern | Major concerns | No concerns | Major concerns | Low | Imprecision, Incoherence |
| Acupunture:MBSR | 0 | No concerns | Low risk | No concern | Major concerns | No concerns | Major concerns | Low | Imprecision, Incoherence |
| Acupunture:SH | 0 | Major concerns | Low risk | No concern | Major concerns | No concerns | Major concerns | Very low | Within-study bias, Imprecision, Incoherence |
| Acupunture:TAU | 0 | No concerns | Low risk | No concern | Major concerns | No concerns | Major concerns | Low | Imprecision, Incoherence |
| Acupunture:dCBTi | 0 | Some concerns | Low risk | No concern | No concerns | No concerns | Major concerns | Low | Within-study bias, Incoherence |
| Acupunture:gCBTi | 0 | No concerns | Low risk | No concern | Major concerns | No concerns | Major concerns | Low | Imprecision, Incoherence |
| Acupunture:sCBTi | 0 | No concerns | Low risk | No concern | Major concerns | No concerns | Major concerns | Low | Imprecision, Incoherence |
| Acupunture:stepcare | 0 | No concerns | Low risk | No concern | Major concerns | No concerns | Major concerns | Low | Imprecision, Incoherence |
| Acupunture:tCBTi | 0 | Some concerns | Low risk | No concern | Major concerns | No concerns | Major concerns | Very low | Within-study bias, Imprecision, Incoherence |
| BT:EX | 0 | Some concerns | Low risk | No concern | Major concerns | No concerns | Major concerns | Very low | Within-study bias, Imprecision, Incoherence |
| BT:MBSR | 0 | Some concerns | Low risk | No concern | Major concerns | No concerns | Major concerns | Very low | Within-study bias, Imprecision, Incoherence |
| BT:SH | 0 | Some concerns | Low risk | No concern | Major concerns | No concerns | Major concerns | Very low | Within-study bias, Imprecision, Incoherence |
| BT:TAU | 0 | Some concerns | Low risk | No concern | Major concerns | No concerns | Major concerns | Very low | Within-study bias, Imprecision, Incoherence |
| BT:dCBTi | 0 | Some concerns | Low risk | No concern | No concerns | No concerns | Major concerns | Low | Within-study bias |
| BT:gCBTi | 0 | Some concerns | Low risk | No concern | Major concerns | No concerns | Major concerns | Very low | Within-study bias, Imprecision, Incoherence |
| BT:sCBTi | 0 | Some concerns | Low risk | No concern | Major concerns | No concerns | Major concerns | Very low | Within-study bias, Imprecision, Incoherence |
| BT:stepcare | 0 | No concerns | Low risk | No concern | Major concerns | No concerns | Major concerns | Low | Within-study bias, Imprecision, Incoherence |
| BT:tCBTi | 0 | Some concerns | Low risk | No concern | Major concerns | No concerns | Major concerns | Very low | Within-study bias, Imprecision, Incoherence |
| EX:MBSR | 0 | No concerns | Low risk | No concern | Major concerns | No concerns | Major concerns | Low | Imprecision, Incoherence |
| EX:SH | 0 | Some concerns | Low risk | No concern | Major concerns | No concerns | Major concerns | Very low | Within-study bias, Imprecision, Incoherence |
| EX:TAU | 0 | No concerns | Low risk | No concern | Major concerns | No concerns | Major concerns | Low | Imprecision, Incoherence |
| EX:dCBTi | 0 | Some concerns | Low risk | No concern | No concerns | No concerns | Major concerns | Low | Within-study bias, Incoherence |
| EX:iCBTi | 0 | No concerns | Low risk | No concern | Major concerns | No concerns | Major concerns | Low | Imprecision, Incoherence |
| EX:stepcare | 0 | No concerns | Low risk | No concern | Major concerns | No concerns | Major concerns | Low | Imprecision, Incoherence |
| EX:tCBTi | 0 | Some concerns | Low risk | No concern | Major concerns | No concerns | Major concerns | Very low | Within-study bias, Imprecision, Incoherence |
| MBSR:SH | 0 | Some concerns | Low risk | No concern | Major concerns | No concerns | Major concerns | Very low | Within-study bias, Imprecision, Incoherence |
| MBSR:TAU | 0 | No concerns | Low risk | No concern | Major concerns | No concerns | Major concerns | Low | Imprecision, Incoherence |
| MBSR:dCBTi | 0 | Some concerns | Low risk | No concern | No concerns | No concerns | Major concerns | Low | Within-study bias, Incoherence |
| MBSR:iCBTi | 0 | No concerns | Low risk | No concern | Major concerns | No concerns | Major concerns | Low | Imprecision, Incoherence |
| MBSR:sCBTi | 0 | No concerns | Low risk | No concern | Major concerns | No concerns | Major concerns | Low | Imprecision, Incoherence |
| MBSR:stepcare | 0 | No concerns | Low risk | No concern | Major concerns | No concerns | Major concerns | Low | Imprecision, Incoherence |
| MBSR:tCBTi | 0 | Some concerns | Low risk | No concern | Major concerns | No concerns | Major concerns | Very low | Within-study bias, Imprecision, Incoherence |
| SH:TAU | 0 | Some concerns | Low risk | No concern | Major concerns | No concerns | Major concerns | Very low | Within-study bias, Imprecision, Incoherence |
| SH:dCBTi | 0 | Some concerns | Low risk | No concern | No concerns | No concerns | Major concerns | Low | Within-study bias, Incoherence |
| SH:gCBTi | 0 | Some concerns | Low risk | No concern | Major concerns | No concerns | Major concerns | Very low | Within-study bias, Imprecision, Incoherence |
| SH:iCBTi | 0 | Major concerns | Low risk | No concern | Major concerns | No concerns | Major concerns | Very low | Within-study bias, Imprecision, Incoherence |
| SH:sCBTi | 0 | Some concerns | Low risk | No concern | Major concerns | No concerns | Major concerns | Very low | Within-study bias, Imprecision, Incoherence |
| SH:stepcare | 0 | Major concerns | Low risk | No concern | Major concerns | No concerns | Major concerns | Very low | Within-study bias, Imprecision, Incoherence |
| TAU:stepcare | 0 | No concerns | Low risk | No concern | Major concerns | No concerns | Major concerns | Low | Imprecision, Incoherence |
| dCBTi:gCBTi | 0 | Some concerns | Low risk | No concern | No concerns | No concerns | Major concerns | Low | Within-study bias, Incoherence |
| dCBTi:iCBTi | 0 | Some concerns | Low risk | No concern | No concerns | No concerns | Major concerns | Low | Within-study bias, Incoherence |
| dCBTi:stepcare | 0 | Some concerns | Low risk | No concern | No concerns | No concerns | Major concerns | Low | Within-study bias, Incoherence |
| dCBTi:tCBTi | 0 | Some concerns | Low risk | No concern | No concerns | No concerns | Major concerns | Low | Within-study bias, Incoherence |
| gCBTi:iCBTi | 0 | Major concerns | Low risk | No concern | Major concerns | No concerns | Major concerns | Very low | Within-study bias, Imprecision, Incoherence |
| gCBTi:sCBTi | 0 | No concerns | Low risk | No concern | Some concerns | Some concerns | Major concerns | Low | Imprecision, Heterogeneity, Incoherence |
| gCBTi:stepcare | 0 | No concerns | Low risk | No concern | Major concerns | No concerns | Major concerns | Low | Imprecision, Incoherence |
| gCBTi:tCBTi | 0 | Some concerns | Low risk | No concern | Major concerns | No concerns | Major concerns | Very low | Within-study bias, Imprecision, Incoherence |
| iCBTi:tCBTi | 0 | Some concerns | Low risk | No concern | Major concerns | No concerns | Major concerns | Very low | Within-study bias, Imprecision, Incoherence |
| sCBTi:stepcare | 0 | No concerns | Low risk | No concern | Major concerns | No concerns | Major concerns | Low | Imprecision, Incoherence |
| sCBTi:tCBTi | 0 | Some concerns | Low risk | No concern | Major concerns | No concerns | Major concerns | Very low | Within-study bias, Imprecision, Incoherence |
| stepcare:tCBTi | 0 | Some concerns | Low risk | No concern | Major concerns | No concerns | Major concerns | Very low | Within-study bias, Imprecision, Incoherence |

Abbreviations: BT = behavioral therapy, dCBTi = digital CBTi, EX = exercise, gCBTi = group CBTi, iCBTi = individual CBTi, ISI = insomnia sleep index, MBSR = mindfulness-based stress reduction, rTMS = repetitive transcranial magnetic stimulation, sCBTi = self-help CBTi, SE = sleep efficiency, SH = sleep hygiene, SOL = sleep onset latency, stepcare = stepped-care CBTi, TAU = treatment as usual, tCBTi = teleCBTi, TST = total sleep time, and WASO = wake after sleep onset

**Supplementary Material 20** The results of forest plots for sleep outcomes

(1) ISI	(2) SOL

(3) WASO	(4) TST

(5) SE

Abbreviations: BT = behavioral therapy, dCBTi = digital CBTi, EX = exercise, gCBTi = group CBTi, iCBTi = individual CBTi, ISI = insomnia sleep index, MBSR = mindfulness-based stress reduction, rTMS = repetitive transcranial magnetic stimulation, sCBTi = self-help CBTi, SE = sleep efficiency, SH = sleep hygiene, SOL = sleep onset latency, stepcare = stepped-care CBTi, TAU = treatment as usual, tCBTi = teleCBTi, TST = total sleep time, and WASO = wake after sleep onset

**Supplementary Material 21** Included articles

1.	Casault, L., et al., A randomized-controlled trial of an early minimal cognitive-behavioural therapy for insomnia comorbid with cancer. Behav Res Ther, 2015. 67: p. 45–54.

2.	Clara, M.I., et al., Web-based cognitive-behavioral therapy for insomnia in cancer survivors: The OncoSleep randomized trial. Sleep Med, 2025. 129: p. 67–74.

3.	Dirksen, S.R. and D.R. Epstein, Efficacy of an insomnia intervention on fatigue, mood and quality of life in breast cancer survivors. J Adv Nurs, 2008. 61(6): p. 664–75.

4.	Espie, C.A., et al., Randomized controlled clinical effectiveness trial of cognitive behavior therapy compared with treatment as usual for persistent insomnia in patients with cancer. J Clin Oncol, 2008. 26(28): p. 4651–8.

5.	Fiorentino, L., et al., Individual cognitive behavioral therapy for insomnia in breast cancer survivors: a randomized controlled crossover pilot study. Nat Sci Sleep, 2010. 2: p. 1–8.

6.	Garland, S.N., et al., Mindfulness-based stress reduction compared with cognitive behavioral therapy for the treatment of insomnia comorbid with cancer: a randomized, partially blinded, noninferiority trial. J Clin Oncol, 2014. 32(5): p. 449–57.

7.	Garland, S.N., et al., Randomized Controlled Trial of Virtually Delivered Cognitive Behavioral Therapy for Insomnia to Address Perceived Cancer-Related Cognitive Impairment in Cancer Survivors. J Clin Oncol, 2024. 42(17): p. 2094–2104.

8.	Garland, S.N., et al., Acupuncture Versus Cognitive Behavioral Therapy for Insomnia in Cancer Survivors: A Randomized Clinical Trial. J Natl Cancer Inst, 2019. 111(12): p. 1323–1331.

9.	Irwin, M.R., et al., Tai Chi Chih Compared With Cognitive Behavioral Therapy for the Treatment of Insomnia in Survivors of Breast Cancer: A Randomized, Partially Blinded, Noninferiority Trial. J Clin Oncol, 2017. 35(23): p. 2656–2665.

10.	Matthews, E.E., et al., Cognitive behavioral therapy for insomnia outcomes in women after primary breast cancer treatment: a randomized, controlled trial. Oncol Nurs Forum, 2014. 41(3): p. 241–53.

11.	Mercier, J., H. Ivers, and J. Savard, A non-inferiority randomized controlled trial comparing a home-based aerobic exercise program to a self-administered cognitive-behavioral therapy for insomnia in cancer patients. Sleep, 2018. 41(10).

12.	Moon, S.Y., et al., Comparative Effectiveness of Cheonwangbosimdan (Tian Wang Bu Xin Dan) Versus Cognitive-Behavioral Therapy for Insomnia in Cancer Patients: A Randomized, Controlled, Open-Label, Parallel-Group, Pilot Trial. Integr Cancer Ther, 2020. 19: p. 1534735420935643.

13.	Myers, J.S., et al., Sleep Hygiene Education, ReadiWatch Actigraphy, and Telehealth Cognitive Behavioral Training for Insomnia for People With Prostate Cancer. Oncol Nurs Forum, 2025. 52(2): p. E15–E34.

14.	Oswald, L.B., et al., Pilot randomized controlled trial of eHealth cognitive-behavioral therapy for insomnia among Spanish-speaking breast cancer survivors. J Behav Med, 2022. 45(3): p. 503–508.

15.	Ritterband, L.M., et al., Initial evaluation of an Internet intervention to improve the sleep of cancer survivors with insomnia. Psychooncology, 2012. 21(7): p. 695–705.

16.	Savard, J., et al., Is a video-based cognitive behavioral therapy for insomnia as efficacious as a professionally administered treatment in breast cancer? Results of a randomized controlled trial. Sleep, 2014. 37(8): p. 1305–14.

17.	Savard, J., et al., Efficacy of a stepped care approach to deliver cognitive-behavioral therapy for insomnia in cancer patients: a noninferiority randomized controlled trial. Sleep, 2021. 44(11).

18.	Savard, J., et al., Randomized study on the efficacy of cognitive-behavioral therapy for insomnia secondary to breast cancer, part I: Sleep and psychological effects. J Clin Oncol, 2005. 23(25): p. 6083–96.

19.	Starling, C.M., et al., Voice-Activated Cognitive Behavioral Therapy for Insomnia: A Randomized Clinical Trial. JAMA Netw Open, 2024. 7(9): p. e2435011.

20.	Tian, S., et al., Pilot randomized controlled trial of transcranial magnetic stimulation for the treatment of insomnia in cancer survivors: An efficacy, safety, and feasibility therapy. Int J Cancer, 2025. 157(6): p. 1055–1063.

21.	Zachariae, R., et al., Internet-Delivered Cognitive-Behavioral Therapy for Insomnia in Breast Cancer Survivors: A Randomized Controlled Trial. J Natl Cancer Inst, 2018. 110(8): p. 880–887.

22.	Zheng XH. Effects of cognitive behavioral therapy on insomnia patients undergoing chemotherapy after breast cancer surgery. Chinese J RuralMed Pharm. 2015;22(21):14-15.
